# A Photoresponsive Hybrid of Viruses and Supramolecular Peptide Fibers for Multidimensional Control of Patterning and Infection

**DOI:** 10.1002/anie.202508528

**Published:** 2025-08-25

**Authors:** Atsuya Yaguchi, Noriyuki Uchida, Daiki Miura, Go Watanabe, Hirotsugu Hiramatsu, Itsuki Ajioka, Teruhiko Matsubara, Toshinori Sato, Chinbat Enkhzaya, Shunto Itani, Tomokazu Saito, Takahiro Muraoka

**Affiliations:** ^1^ Department of Applied Chemistry Graduate School of Engineering Tokyo University of Agriculture and Technology 2‐24‐16 Naka‐cho Koganei Tokyo 184–8588 Japan; ^2^ Department of Physics School of Science Kitasato University 1‐15‐1 Kitasato Sagamihara, Minami‐ku 252–0373 Japan; ^3^ Project of Regenerative Medicine for Stroke using Supramolecular Peptide Kanagawa Institute of Industrial Science and Technology (KISTEC) 3‐2‐1 Sakato, Takatsu‐ku Kawasaki Kanagawa 213‐0012 Japan; ^4^ Department of Applied Chemistry and Institute of Molecular Science National Yang Ming Chiao Tung University 1001 Ta‐Hsueh Road Hsinchu 30010 Taiwan; ^5^ Center for Brain Integration Research (CBIR) Institute of Integrated Research, Institute of Science Tokyo 1‐5‐45 Yushima Bunkyo‐ku Tokyo 113–8510 Japan; ^6^ Research Center for Autonomous Systems Materialogy (ASMat) Institute of Integrated Research Institute of Science Tokyo 4259 Nagatsuta‐cho, Midori‐ku Yokohama Kanagawa 226–8501 Japan; ^7^ Department of Material‐based Neuroscience Graduate School of Medical and Dental Sciences Institute of Science Tokyo 1‐5‐45 Yushima Bunkyo‐ku Tokyo 113–8510 Japan; ^8^ Department of Biosciences and Informatics, Faculty of Science and Technology Keio University Yokohama Kanagawa 223–8522 Japan

**Keywords:** Gels, Peptides, Polymers, Supramolecular chemistry, Viruses

## Abstract

Viruses are versatile colloidal materials in their biofunctions, monodispersed and periodic structures, and high surface designability. For expanding the applicability of virus‐based materials, spatiotemporally controlled immobilization and dispersion of viruses with retained activity should be useful, though control of the dynamic nature of viruses hybridized with commonly used polymers has been difficult due to their strong interactions. Here, we report a self‐assembling peptide (A2Az) enabling photo control of adhesion and dispersion of M13 bacteriophage virus (M13 phage) and successfully demonstrate patterning of localization and infection of the virus. A2Az is a cationic peptide with amphiphilicity that consists of eight amino acid residues containing a photo‐responsive azobenzene group at the second position and self‐assembles into a helical supramolecular fiber to form a hydrogel. The helical fibrillar morphology of A2Az exhibits strong interaction with M13 phage, allowing for immobilization not only on a two‐dimensional surface but also in a three‐dimensional hydrogel with suppression of infectivity. The A2Az fiber undergoes a light‐triggered fiber‐to‐particle transition and releases the immobilized M13 phage with retained infectivity for the photo‐controlled patterning of localization and infection. This approach has potential applicability to various virus‐based biomaterials, such as structural materials and materials for photo‐selective gene transfection to cells.

## Graphical abstract

A hybrid of a bacteriophage virus and a helical supramolecular fiber, composed of an amphiphilic peptide containing an azobenzene group (A2Az), as a virus hybridizable self‐assembling peptide is reported. The hybrid undergoes disassembly by photo‐induced depolymerization of the A2Az fiber, which enables site‐selective control of the adhesion and infection behaviors of the bacteriophage not only on 2D substrates but also in 3D hydrogel environments.

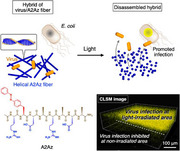

## Introduction

Viruses, formed by the assembly of capsid proteins, are useful as biomaterials because of their variety of morphologies with structural periodicity and surface designability. In particular, hybrids of viruses and polymers^[^ ^1^, ^2^, ^3^, ^4^, ^5^, ^6^, ^7^, ^8^, ^9^, ^10^, ^11^, ^12^ ^]^ have attracted attention not only for medical applications as drug carriers for gene therapy^[^ ^5^ ^]^ but also as nm‐ and µm‐size building blocks to construct structural materials for electronic devices such as lithium‐ion batteries^[^ ^11^ ^]^ and piezoelectric materials.^[^ ^12^ ^]^ However, it has been difficult to dynamically control such virus‐polymer hybrids due to their strong multivalent interactions, limiting patterning of the virus‐based materials required for advanced applications.^[^ ^13^, ^14^, ^15^ ^]^ Although photo control of virus‐polymer hybrid colloids in solution using light‐responsive cationic dendrimers^[^ ^4^ ^]^ has been reported, controls of the dispersibility of virus‐polymer hybrids in two‐ dimensional (2D) and three‐dimensional (3D) networks have not been achieved.

**Figure 1:**
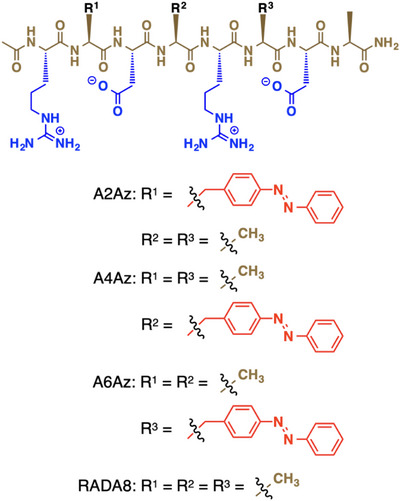
Molecular structures of A2Az, A4Az, and A6Az containing a *trans*‐form of the azobenzene (Az) group, and RADA8.

**Figure 2:**
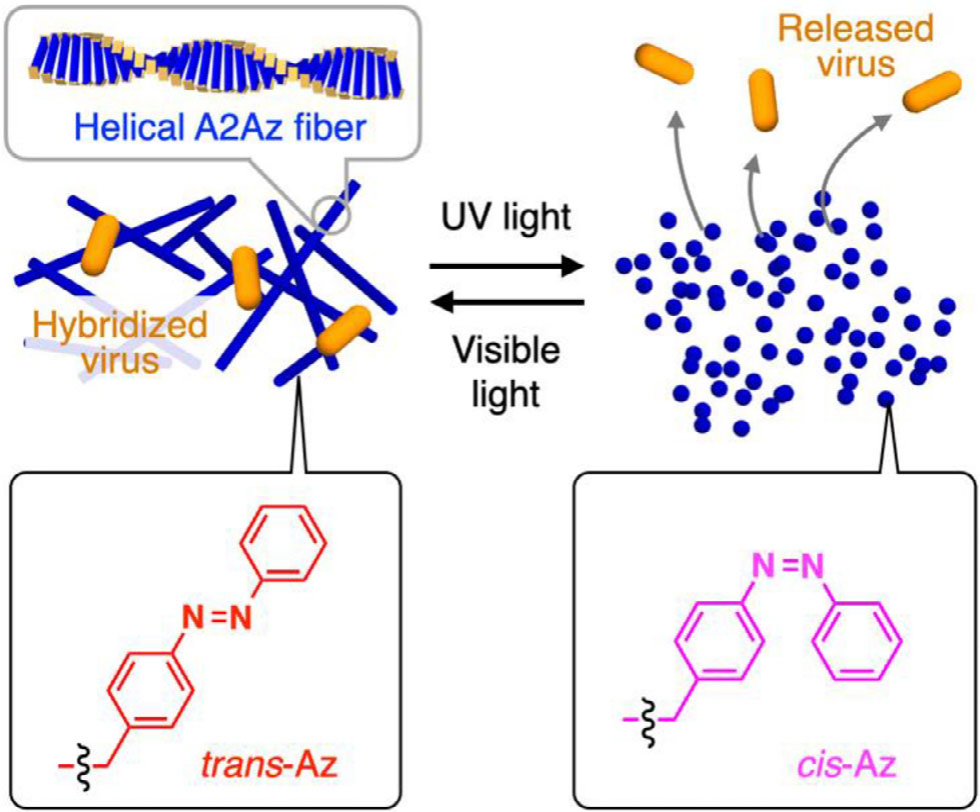
Schematic illustration of the concept of the photoresponsive virus and A2Az hybrid. The helical A2Az fiber effectively hybridizes with the virus, which is then released by UV‐induced depolymerization of the A2Az fiber by *trans*‐to‐*cis* isomerization of the Az group.

Here, we developed a self‐assembling peptide (Figure 1, A2Az; Ac‐RAzDARADA‐NH_2_) consisting of eight amino acid residues containing an amino acid residue with an azobenzene (Az) side chain. A2Az self‐assembles into helical supramolecular nanofibers in aqueous media to form a hydrogel, and subsequent UV light irradiation prompts a fiber‐to‐particle morphological change of A2Az accompanied by a gel‐to‐sol transition. M13 bacteriophage virus (M13 phage),^[^ ^6^, ^7^, ^8^, ^9^, ^10^, ^11^, ^16^, ^17^, ^18^, ^19^, ^20^, ^21^ ^]^ which has been utilized for the development of various functional biomaterials by exploiting its unique rod shape (with a length of 1 µm) and ease of manipulation, is adhered onto the A2Az fibers and its infectivity is suppressed temporally by the adhesion. Using the virus and peptide fiber hybrid, we demonstrate the first example of site‐selective control of the adhesion of M13 phage on a 2D substrate and its infection event in a 3D hydrogel environment by the light‐triggered fiber‐to‐particle transition of A2Az, allowing for virus dispersion and recovery of virus infectivity (Figure 2).

## Results and Discussion

### Design of Photoresponsive Self‐Assembling Peptides

**Figure 3:**
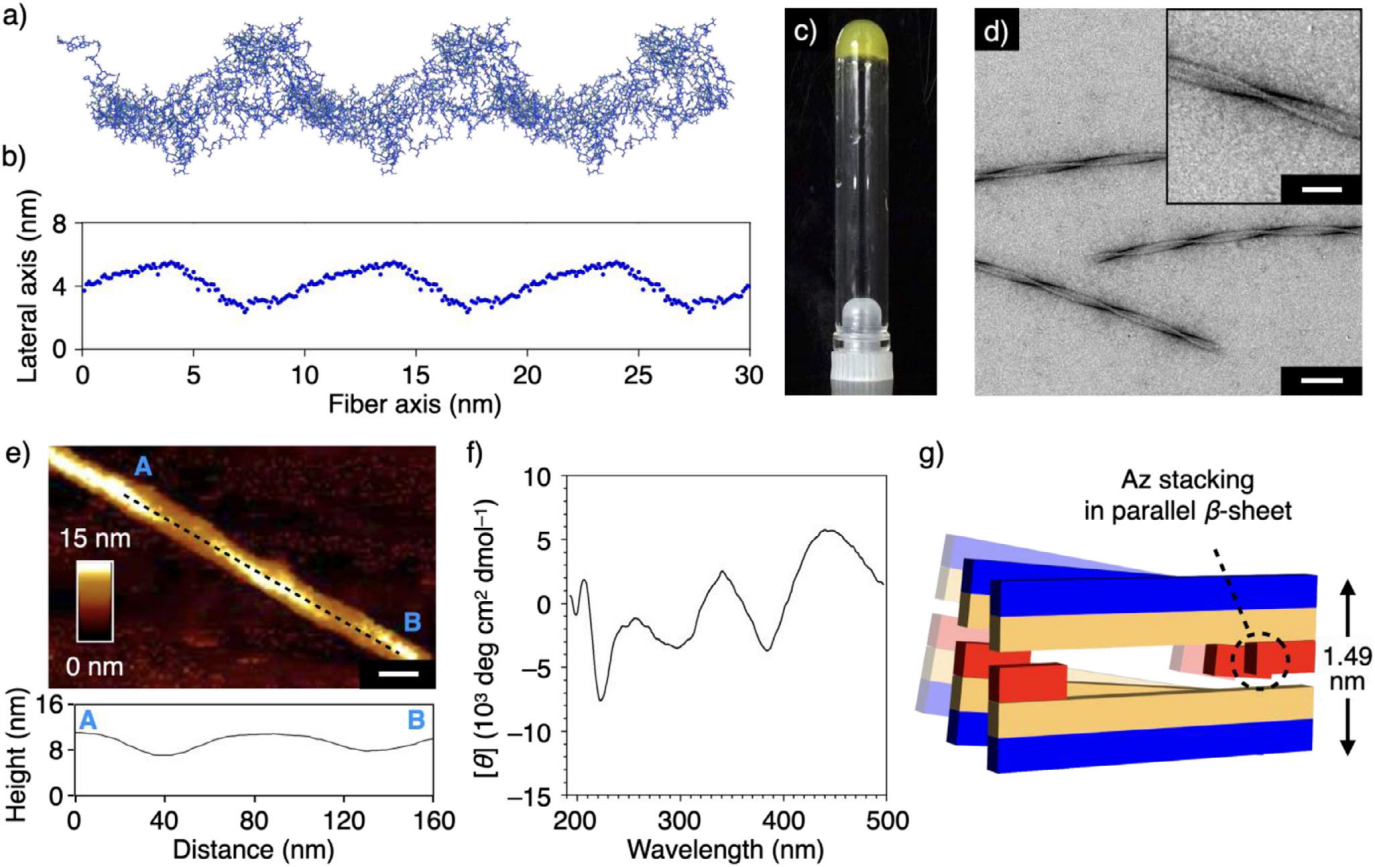
a) and b) Snapshot (a) and distribution of the particle center of mass (b) of the supramolecular structure of the A2Az fiber in water at 300 K, as calculated by all‐atom molecular dynamics (MD) simulations. c) Photograph of the hydrogel of the A2Az fiber (1.0 wt%) containing trifluoroacetic acid (TFA; 2.2 v/v%) at 20 °C. d) Transmission electron micrograph (TEM) of the A2Az fibers. The inset shows a higher magnification image. Scale bars: 100 and 50 nm (inset). Samples were negatively stained with uranyl acetate. e) Atomic force microscopy (AFM) image with height profile of the A2Az fiber. Scale bar: 20 nm. Dashed line indicates the region of the height profile. f) Circular dichroism (CD) spectrum of the A2Az fiber (1.0 wt%) in water containing TFA (2.2 v/v%) at 20 °C. g) Plausible Az stacking in parallel *β*‐sheets. The hydrophilic region, the hydrophobic region, and the Az group of A2Az are highlighted as blue, cream, and red, respectively.

For the design of nanofibers with stimuli‐responsive assembly and disassembly capability, supramolecular peptide fibers have attracted increasing attention. Peptides have advantages over other types of molecules in terms of structural designability through established solid‐phase synthesis and the capability of forming a variety of higher‐order structures. Self‐assembling peptides are one of the important classes of biomaterials that form supramolecular fibers in physiological conditions and are advantageous for constructing stimuli‐responsive transformable systems by adjusting noncovalent interactions. As represented by RADA16 (Ac‐RADARADARADARADA‐NH_2_), amphiphilic peptides consisting of iterative amino acid sequences between hydrophilic and hydrophobic residues can self‐assemble into *β*‐sheet motifs through hydrophobic interactions and hydrogen bonds, leading to self‐assembly into nanofibers to form hydrogels.^[^ ^22^, ^23^, ^24^ ^]^ To construct photoresponsive systems, we designed a series of peptides based on RADA8 (Figure 1, Ac‐RADARADA‐NH_2_), in which one alanine residue was replaced by Az at 2‐, 4‐, or 6‐position (Figure 1, A2Az; Ac‐RAzDARADA‐NH_2_, A4Az; Ac‐RADAzRADA‐NH_2_, and A6Az; Ac‐RADARAzDA‐NH_2_, respectively). When we performed molecular dynamics (MD) simulation for A2Az, A4Az, and A6Az, they showed formation of one‐dimensional assemblies in silico even with a shorter length than RADA16. Interestingly, the MD simulation of A2Az assembly predicted the formation of a winding helical fiber, while the calculation of A4Az and A6Az assemblies resulted in nonhelical fibrillar formation. The center‐of‐mass distribution mapping of A2Az assemblies calculated by MD simulation showed a wavy profile characteristic of a helical alignment, while those of A4Az and A6Az fibrils showed profiles consisting of less wavy lines (Figures 3a,b and S1).

### Characterization of Peptide Self‐Assemblies

**Figure 4:**
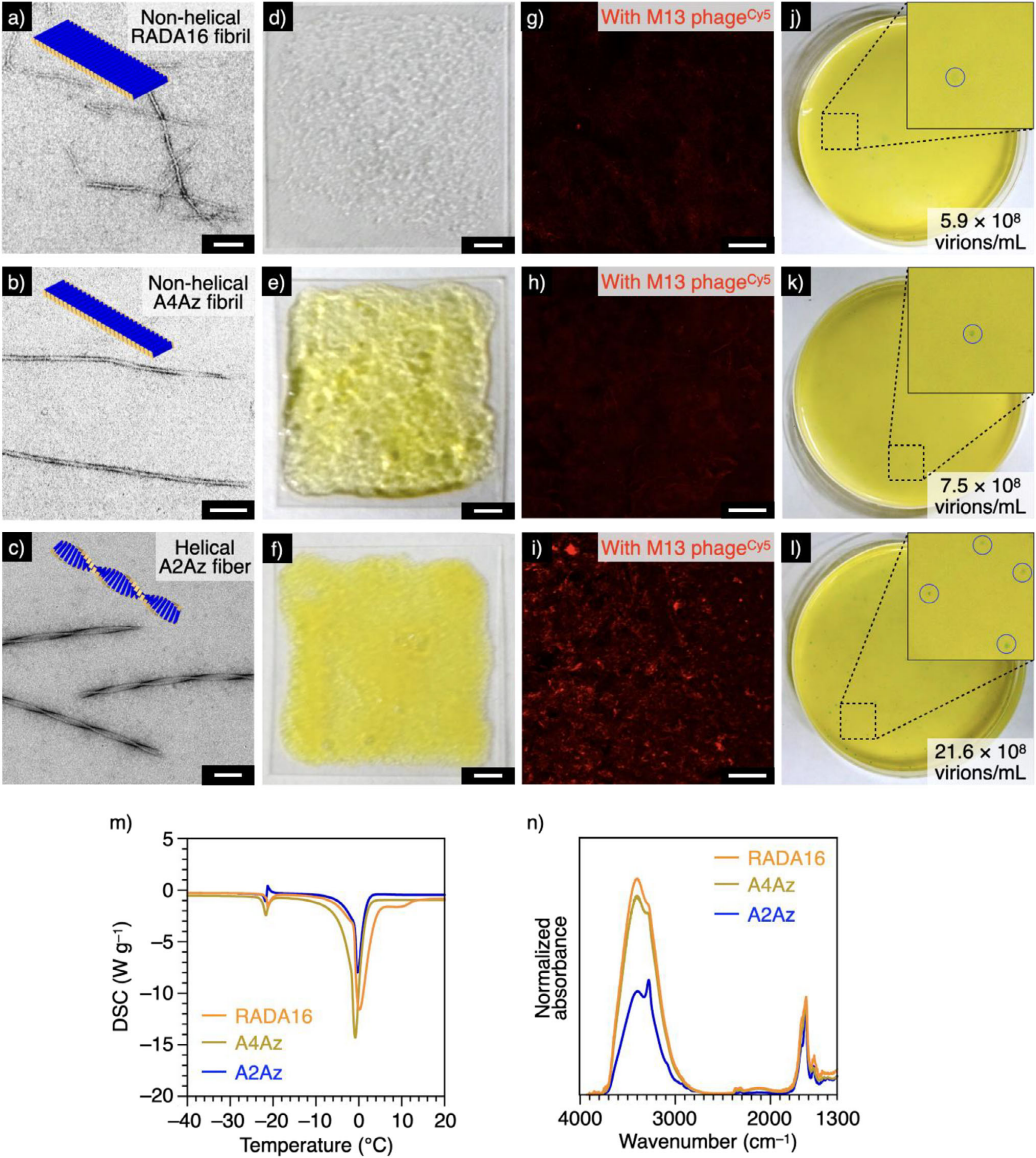
a)–c) TEM images of RADA16 fibrils (a), A4Az fibrils (b), and A2Az helical fibers (c). Scale bars: 100 nm. d)–f) Photographs of glass substrates coated with RADA16 fibril (d), A4Az fibril (e), and A2Az fiber (f). Scale bars: 2 mm. g)–i) Confocal laser scanning microscopic (CLSM) images (*λ*_ex_ = 647 nm, *λ*_obs _ = 650–750 nm) of the glass substrates coated with RADA16 fibril (g), A4Az fibril (h), and A2Az fiber (i) after incubation with Cy5‐labled M13 phage (M13 phage^Cy5^, 17.9 × 10^11^ virions mL^−1^). Scale bars: 100 µm. j)–l) Photographs of 5‐bromo‐4‐chloro‐3‐indolyl *β*‐D‐galactopyranoside (X‐gal) plates after plaque assays for M13 phage adsorbed to RADA16 fibril‐(j), A4Az fibril‐(k), and A2Az fiber‐coated glass substates (l). Insets: magnified images of the X‐gal plates; plaques of M13 phage‐infected *E. coli* are indicated by blue circles. m) Differential scanning calorimetry (DSC) traces of 1.0 wt% hydrogel samples of RADA16 fibrils (orange), A4Az fibrils (brown), and A2Az fibers (blue). n) Infrared (IR) spectra of 1.0 wt% hydrogel samples of RADA16 fibrils (orange), A4Az fibrils (brown), and A2Az fibers (blue).

Considering the MD simulation results, we investigated the self‐assembling behavior of the peptides. Upon incubation of A2Az, A4Az, and A6Az at peptide concentrations of 1.0 wt% in an aqueous medium containing 2.2 v/v% trifluoroacetic acid (TFA)^[^ ^24^ ^]^ at 20 °C, A2Az and A4Az turned into nonfluidic states within 2 min, while A6Az remained a fluidic suspension (Figures 3c and S2 and S3). Rheological measurements indicated hydrogel formation of A2Az and A4Az, as demonstrated by the fact that the storage moduli (*G*’) of these peptide samples were larger than the loss moduli (*G*″) (Figure S4), and these hydrogels showed a thixotropic nature (Figure S5).^[^ ^25^ ^]^ Interestingly, transmission electron microscopic (TEM) imaging of A2Az revealed the formation of helical fibers (Figure 3d). The helix formation of A2Az with a repeating height profile ranging from 1 to 5 nm was visualized by atomic force microscopic (AFM) observation (Figure 3e). In contrast, A4Az formed nonhelical fibrils when observed by TEM and AFM (Figures 4b and S2), which was analogous to the RADA16 fibrils (Figure 4a), and A6Az formed plate‐like objects (Figure S3). Ac‐RAzDA‐NH_2_ consisting of only four residues formed nonfibrous fragmental structures (Figure S6). These results indicated that the molecular length of the peptides and the position of the bulky Az group are critical factors, not only for fiber formation but also for helical twisting of the fiber. Indeed, the incorporation of other bulky aromatic side groups, such as naphthyl and biphenyl groups, instead of the Az group also led to the formation of helical fibers (Figure S7); in contrast, replacing the Az group in A4Az with naphthyl and biphenyl groups resulted in the formation of nonhelical fibrils (Figure S8). Circular dichroism (CD) spectroscopic measurement of A2Az revealed a split pattern in the absorption region of the Az unit showing negative and positive Cotton effects at 385 and 448 nm, respectively, suggesting helical assembly of the Az moieties (Figures 3f and S9).^[^ ^26^ ^]^ In contrast, A4Az exhibited a distinctly different CD pattern (Figure S9). Lyophilized samples of A2Az and A4Az hydrogels also showed distinct reflectance spectral profiles (Figure S10). In addition, to reduce the degree of Az stacking in the A2Az fibers, we mixed A2Az with RADA8, which exhibited limited self‐assembly (Figure S11), and observed that the mixture (weight ratio of A2Az:RADA8 = 80:20, 1.0 wt% in total) afforded nonhelical fibers (A2Az/RADA8 fiber) as observed by TEM and AFM observations, CD spectroscopic measurement, and viscoelasticity analysis (Figure S12). These results suggest that the geometry of the Az stacking is associated with the formation of helical fibers of A2Az. Furthermore, when we evaluated the packing structure of the peptide self‐assemblies by infrared (IR) spectra, A2Az and A4Az exhibited characteristic sharp vibrational bands at 1622–1626 cm^−1^ due to *β*‐sheet structures (Figure S13); the analysis of isotope‐labeled samples revealed that both peptides formed parallel *β*‐sheet structures (Figure S14). Zeta potential and ion conductivity profiles of A2Az were essentially identical to those of A4Az (Figure S15). On the other hand, powder X‐ray diffraction (PXRD) analysis showed that the distance between the stacked pair of peptide chains, generated via intramolecular hydrophobic interactions within the A2Az fibers (1.49 nm, Figures 3g and S16), was larger than that of the A4Az fibrils (1.34 nm, Figure S17). These results suggested that the helical morphology of A2Az assemblies might be constructed to decrease the exposure of the hydrophobic portions of the peptide molecules residing at the edge. Azobenzene has been utilized as a photo‐switching unit to develop photoresponsive supramolecular peptide fibers.^[^ ^27^, ^28^ ^]^ In the previous examples, the Az unit was attached at an edge or in the middle of the peptide main chain. In contrast, A2Az and A4Az possess the Az unit at the side chain. The fiber formation of A2Az and the peptides possessing naphthyl and biphenyl units at the 2‐position indicates that the attachment of a bulky aromatic unit at the side chain of an amino acid residue near a terminus is broadly effective for inducing the helical assembly of amphiphilic peptides.

### Hybridization of Peptide Fibers with M13 Phage

Interestingly, helical A2Az fibers showed stronger adhesivity to M13 phage virus than other nonhelical peptide fibers. To evaluate the adhesivity, we first incubated an aqueous suspension of fluorescent Cy5‐labled M13 phage (M13 phage^Cy5^, 17.9 × 10^11^ virions mL^−1^) on a glass substrate coated with nonhelical RADA16 fibrils (Figure 4a,d) for 90 min at 25 °C, followed by washing with Tris‐HCl buffer (1 M Tris‐HCl, pH 7.5). Under confocal laser scanning microscopy (CLSM), we observed fluorescence corresponding to M13 phage^Cy5^ adhered onto the fibrils, although the overall intensity was rather weak (Figure 4g). To evaluate the infection ability of the adhered M13 phage, non Cy5‐labeled M13 phage (17.9 × 10^11^ virions mL^−1^) was adsorbed to the RADA16 fiber‐coated substrate, and we performed a plaque assay using *Escherichia coli* (*E. coli*) cells after eluting the adhered M13 phage with glycine buffer (200 mM glycine, pH 2.2). Since *E. coli* cells infected by M13 phage express *β*‐galactosidase that degrades 5‐bromo‐4‐chloro‐3‐indolyl *β*‐D‐galactopyranoside (X‐gal) to produce a blue indigo dye, the degree of M13 phage infection can be quantified based on the number of spots (plaques) that appear on X‐gal‐containing hydrogel plates (Figure S18).^[^ ^29^ ^]^ The results revealed that M13 phage maintained its infection ability and the amount of M13 phage infection was (5.9 ± 3.3) × 10^8^ virions mL^−1^ (mean ± SD, Figure 4j). When we conducted the same experiment using a substrate coated with nonhelical A4Az (Figure 4b,e) or A2Az/RADA8 fibrils (Figure S19, A2Az:RADA8, weight ratio 80:20), we observed M13 phage^Cy5^‐derived fluorescence of a similar intensity as in the case of RADA16 fibers in CLSM (Figure 4g,h and S19), and no significant difference in the plaque assay ((7.5 ± 2.7) × 10^8^ (mean ± SD) for A4Az, (11.2 ± 3.7) × 10^8^ virions mL^−1^ (mean ± SD) for A2Az/RADA8, respectively, Figures 4k and S19). In contrast, M13 phage showed significantly stronger adhesion to the substrate coated with A2Az fibers (Figures 4c,f and S20) as evaluated by CLSM (Figure 4i) and the plaque assay (Figure 4l, (21.6 ± 2.6) × 10^8^ virions mL^−1^ (mean ± SD)). The stronger adhesion of M13 phage to the A2Az helical fiber‐coated substrate than to the substrates coated with nonhelical peptide fibrils was also indicated by enzyme‐linked immunosorbent assay (Figure S21). In differential scanning calorimetry (DSC) analysis^[^ ^30^, ^31^ ^]^ of A2Az (Figure 4m, blue) to investigate the surface profile of the assembly, we observed the presence of a peak at approximately 0 °C (*ΔH* = 0.90 kJ g^−1^), which was derived from the melting of water molecules in the hydrogel. Notably, this peak was smaller than that observed for the other peptides composed of RADA16 (Figure 4m, orange, *ΔH* = 2.00 kJ g^−1^), A4Az (Figure 4m, brown, *ΔH* = 2.10 kJ g^−1^), and A2Az/RADA8 (Figure S19, *ΔH* = 1.92 kJ g^−1^) forming nonhelical fibrils. Moreover, the IR spectrum of A2Az exhibited a peak in the region of wavenumber *ν*_OH _= 2600–3800 cm^−1^ (Figure 4n, blue) including vibrational signals corresponding to O─H stretching of hydrated water molecules bound to macromolecules.^[^ ^31^, ^32^ ^]^ The peak at *ν*_OH_ exhibited a normalized intensity of 5.3 based on the amide‐derived peaks in the region of *ν*_Amide _= 1500–1800 cm^−1^. On the other hand, the normalized intensities of the hydration peaks at *ν*_OH_ in assemblies of RADA16, A4Az, and A2Az/RADA8 were 7.6 (Figure 4n, orange), 8.2 (Figure 4n, brown), and 7.6 (Figure S19), respectively; these values exceeded that of A2Az. The melting enthalpy of water in DSC analysis is known to change depending on the amount of water molecules strongly binding to the polymer or on the hydration behavior in hydrogels.^[^ ^32^ ^]^ Another study has reported that the proportion of the hydration‐derived peak in IR analysis (*ν* = 2600–3800 cm^−1^) increases depending on the hydration of the polymer.^[^ ^30^, ^31^ ^]^ Therefore, it is likely that the amount of water molecules hydrated on the surface of the helical A2Az fiber would be smaller than those on the nonhelical fibrils. In the MD simulation, it is shown that the helical A2Az fiber contains clustering of the Az units, while the Az units are dispersed throughout the entire area of the nonhelical A4Az fibril (Figure S22). It is also demonstrated that the helical A2Az fibers show stronger adhesion to M13 phage than the A4Az fibrils upon changing the viscoelastic properties of the hydrogels and at an elevated temperature (Figure S23). Furthermore, the adhesion amounts of M13 phage onto the A2Az fibers were hardly affected by decreasing the salt concentration (1 mM Tris‐HCl) or adding urea (9 M), which can enhance electrostatic interactions and hinder hydrogen bonding, respectively (Figure S24). In contact angle measurements, we found that A2Az fiber‐coated substrate has a more hydrophobic surface than the substrates coated by other fibrils (Figure S25). These results suggest that the higher adhesion of helical A2Az fibers to the viruses may reflect a lower energy barrier for the dehydration process required in the adhesive process; therefore, the hydrophobic effect of A2Az fibers likely contributes to the adhesion to M13 phage as the primary factor.^[^ ^33^ ^]^ This was supported by the fact that the helical A2Az fibers also adhere strongly to lysozyme, a cationic protein having surface charge properties opposite to that of M13 phage, than do other nonhelical fibrils (Figure S26). Therefore, we focused on A2Az as a virus‐hybridizable self‐assembling peptide for further investigations. It should be noted that the helical fibers constructed by the peptides possessing naphthyl and biphenyl groups also showed effective adhesion properties to M13 phage, suggesting the broad efficacy of the helical peptide fibers containing bulky aromatic groups on the adhesion (Figure S27).

### Light‐Induced Depolymerization of A2Az Fiber

**Figure 5:**
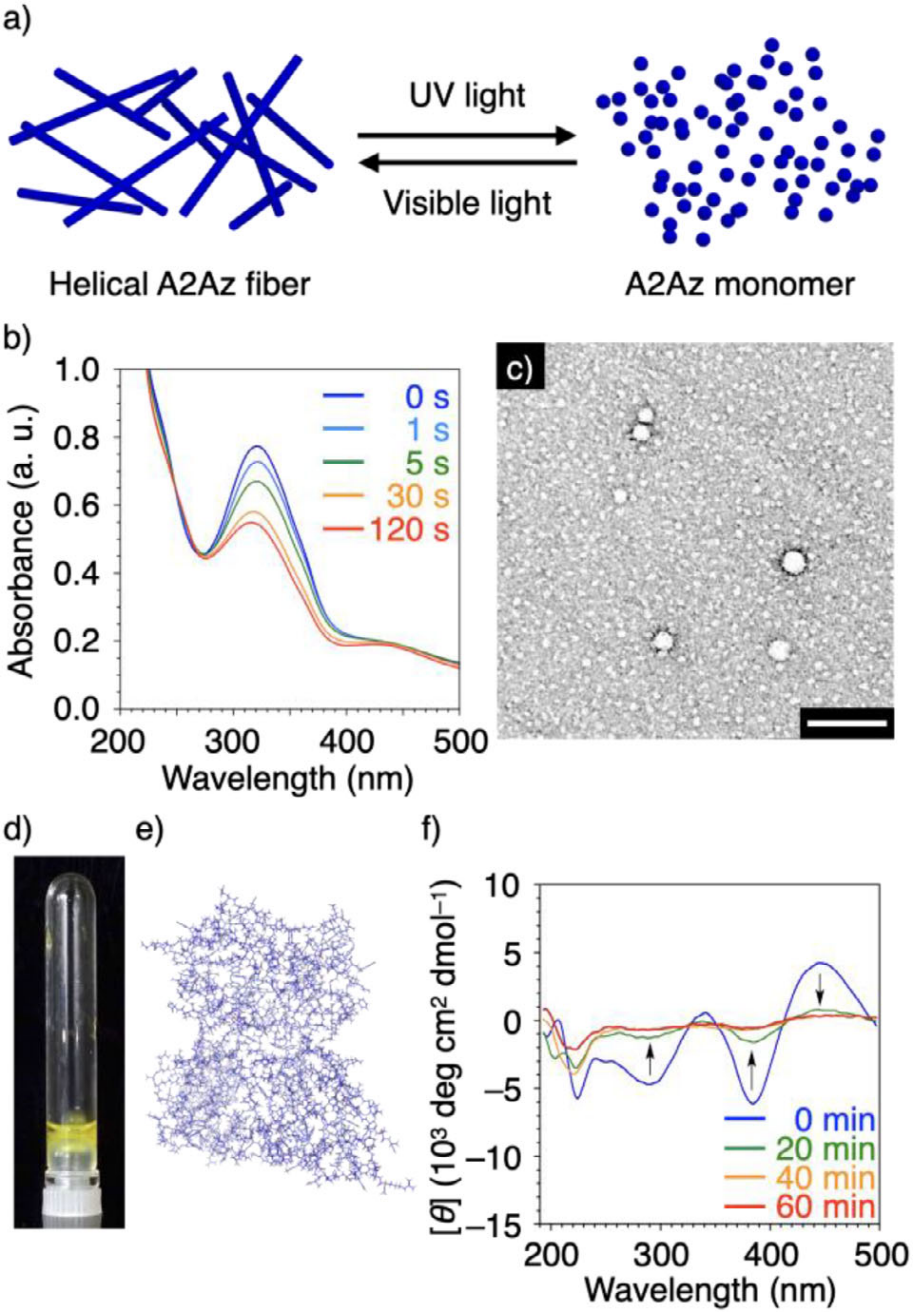
a) Schematic illustration of the transition of A2Az helical fiber to the monomer by UV‐induced isomerization of the Az group. b) UV–vis absorption spectral change of A2Az (0.7 wt% in water containing TFA (2.2 v/v%)) upon irradiation with 350‐nm light for 0 s (blue), 1s (light blue), 5 s (green), 30 s (orange), and 120 s (red). c) TEM image of A2Az (0.7 wt%) in water containing TFA (2.2 v/v%) after irradiation with 350‐nm light for 20 min. Scale bar: 100 nm. The TEM sample was stained with uranyl acetate. d) Photograph of the dispersion of A2Az (0.7 wt%) after irradiation with 350‐nm light for 20 min. e) Snapshot of the supramolecular structure of A2Az containing the *cis*‐form of the Az group in water at 300 K, as calculated by all‐atom MD simulation. f) CD spectral change of A2Az (0.7 wt% in water containing TFA (2.2 v/v%)) upon irradiation with 350‐nm light for 0 min (blue), 20 min (green), 40 min (orange), and 60 min (red). Arrows indicate the directions of spectral change.

A2Az underwent depolymerization by utilizing photo‐isomerization of the Az group (Figure 5a). When the A2Az hydrogel (0.7 wt%) was irradiated with UV light (*λ* = 350 nm), the absorption intensity at 327‐nm peak decreased, while the absorption at 425‐nm peak intensified, indicating *trans*‐to‐*cis* isomerization of the Az group (Figure 5b). Notably, TEM observation of A2Az containing the *cis*‐form of the Az group (*cis*‐A2Az) after UV irradiation showed spherical particles, and a macroscopic gel‐to‐sol phase transition occurred (Figure 5c,d). In MD simulation, *cis*‐A2Az formed a nonfibrillar aggregated structure (Figure 5e), and CD spectroscopic measurement showed a decrease in the Cotton signals of A2Az over the entire wavelength region upon UV irradiation (Figure 5f). IR spectroscopic measurement showed that *cis*‐A2Az contained non‐*β*‐sheet structures, *α*‐helix and/or coils, as its main secondary structures (Figure S28). Furthermore, when 450‐nm visible light was irradiated to depolymerized *cis*‐A2Az to induce backward isomerization of the Az group, A2Az recovered its gel state (Figure S29). As a comparison, we investigated the photo‐responsiveness of A4Az fibril and confirmed that A4Az also showed reversible depolymerization and gelation under the same conditions (Figure S30). These results suggested that the decreased hydrophobicity and the bent conformation of *cis*‐Az, both of which should be less favorable for stacking relative to the *trans*‐form, caused the dissociation of Az stacking and depolymerization.^[^ ^34^, ^35^ ^]^

### Two‐Dimensional Control of Virus Adhesion and Desorption

**Figure 6:**
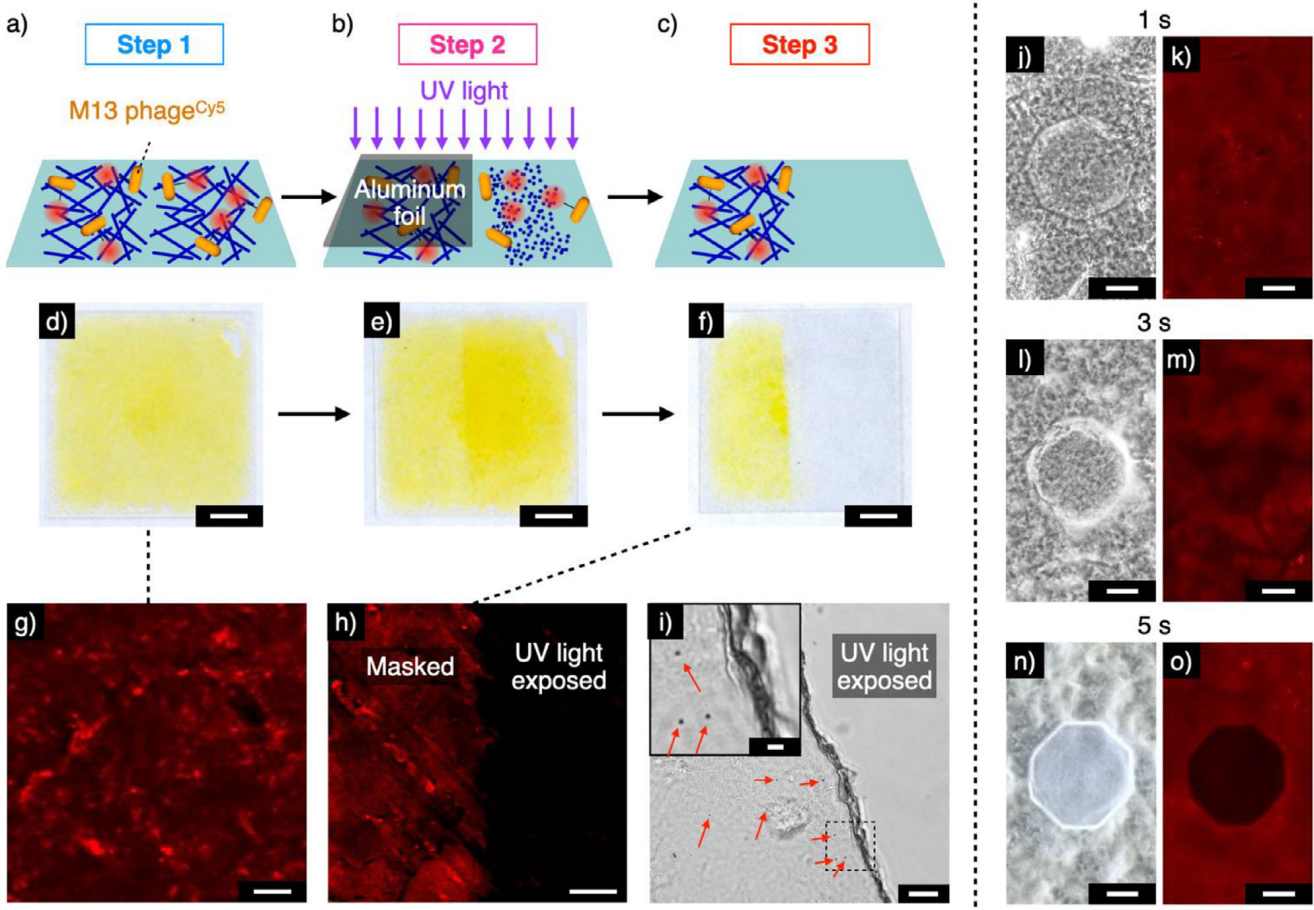
a)–c) Schematic illustrations of photo‐selective dissociation of M13 phage^Cy5^/A2Az‐coated glass substrate. Step 1: Adhesion of M13 phage^Cy5^ to the A2Az‐coated glass substrate (a), Step 2: Ultraviolet (UV) light (*λ* = 350 nm, Xenon lamp) irradiation of the M13 phage^Cy5^‐adhered glass substrate half‐masked with aluminum foil for 40 min (b), Step 3: Rinse of M13 phage^Cy5^ on the glass substrate (c). d)–f) Photographs of the glass substrate at Step 1 (d), Step 2 (e), and Step 3 (f). g) and h) CLSM (*λ*_ex_ = 647 nm, *λ*_obs _= 650–750 nm) images of the glass substrate in Step 1 (g) and Step 3 (h). Scale bars: 50 µm. i) Phase‐contrast image of A2Az‐coated glass substrate containing gold nanoparticles hybridized with gold‐binding M13 phage (M13 phage^Au^) after site‐selective UV light irradiation (*λ* = 370 nm, U‐HGLGPS lamp). The inset shows a higher magnification image of the area of dashed square. Scale bars: 10 and 2 µm (inset). j)–o) Phase‐contrast microscopic (j,l,n) and CLSM (*λ*_ex_ = 647 nm, *λ*_obs_= 650–750 nm) images (k,m,o) of M13 phage^Cy5^‐adhered glass substrate after site‐selective UV light irradiation for 1 (j,k), 3 (l,m), and 5s (n,o) (*λ* = 370 nm, U‐HGLGPS lamp). Scale bars: 100 µm.

Adhesion and desorption behavior of M13 phage^Cy5^ on a 2D substrate can be controlled in a site‐selective manner by utilizing the photo‐responsiveness of A2Az. Fluorescence‐labelled M13 phage^Cy5^ (17.9 × 10^11^ virions mL^−1^) was adhered onto an A2Az‐coated substrate (Figure 6a,d, and g), and a half area of the substrate was masked with aluminum foil (Figure 6b,e). After the sample was irradiated with 350‐nm light for 40 min at 25 °C, the substrate was washed with Tris‐HCl buffer (1 M Tris‐HCl, pH 7.5). As expected, while A2Az in the masked area remained after the process, A2Az in the light‐exposed area was removed selectively (Figure 6c,f). Interestingly, under CLSM observation, fluorescence corresponding to the presence of M13 phage^Cy5^ was observed only in the photo‐masked area (Figure 6h), suggesting that the light‐triggered depolymerization of A2Az fiber facilitated the detachment of M13 phage^Cy5^. When focused UV‐light (*λ* = 370 nm) was irradiated under a microscope, we observed that both A2Az and M13 phage^Cy5^ were gradually dissociated depending on the irradiation time (Figure 6j–o). We also confirmed that the rod‐like structure of M13 phage^Cy5^ was maintained even after the detachment, as visualized by TEM observation (Figure S31). When we utilized M13 phage displaying gold‐binding peptides (M13 phage^Au^), gold nanoparticles can be deposited onto the photo‐patterned A2Az‐coated substrate, suggesting the possible applications to functional optical materials (Figures 6i and S32).^[^ ^36^, ^37^ ^]^ Notably, more complicated patterning, such as a stripe pattern and a micrometer‐scale dot pattern (Figure S33), could be successfully prepared by using a striped photomask or irradiating focused UV‐light (*λ* = 370 nm) with a diameter of 130 µm.

### Three‐dimensional Control of Virus Infection

**Figure 7:**
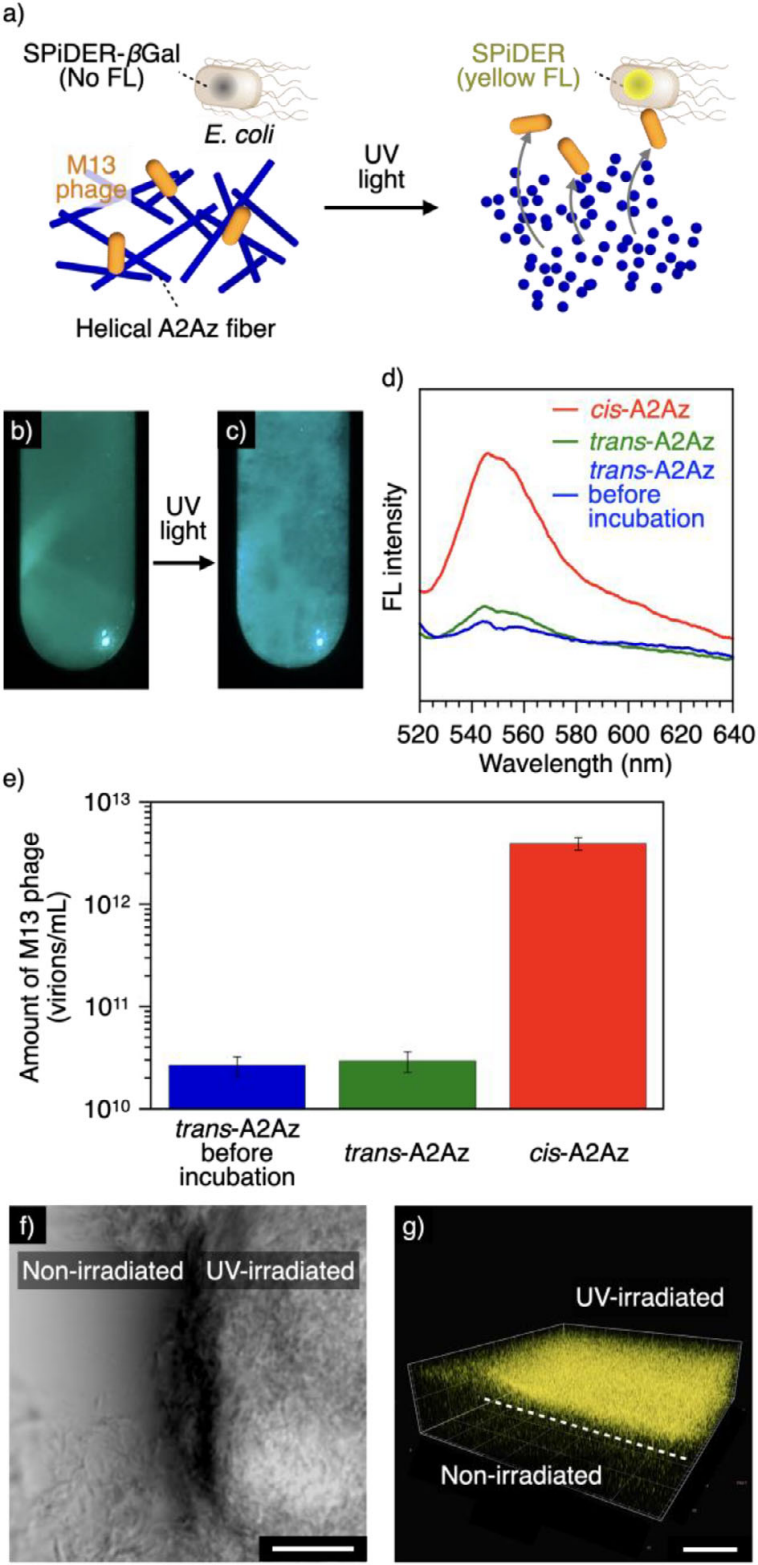
a) Schematic illustration of the photo‐induced infection of M13 phage in A2Az hydrogel. Photographs of an A2Az (0.4 wt%) hydrogel containing M13 phage (1.3 × 1011 virions mL^−1^), *E. coli* cells, and SPiDER‐βGal (0.6 µg mL^−1^) under 500‐nm excitation light b) before and c) after 350‐nm light irradiation (Xenon lamp) for 30 s followed by incubation for 7 days at 37 °C. d) Fluorescent spectra (*λ*_ex_ = 500 nm) of the nonirradiated hydrogel before (blue curve) and after (green curve) incubation, and the hydrogel irradiated with 350‐nm light (Xenon lamp) for 30 s followed by incubation for 7 days at 37 °C (red curve). e) The amount of M13 phage evaluated by the plaque assay in A2Az hydrogel before (blue) and after (green) 7‐day incubation, and after incubation following the 350‐nm light (Xenon lamp) irradiation for 30 s (red). f) and g) Bright‐field image (f) and three‐dimensional CLSM image (*λ*_ex_ = 488 nm, *λ*_obs _= 500–650 nm) of SPiDER dye‐associated fluorescence g) of an A2Az (0.5 wt%) hydrogel containing M13 phage (1.3 × 10^11^ virions mL^−1^) and *E. coli* cells; fluorescence (yellow) was observed exclusively in the region where the hydrogel had been irradiated with 370‐nm light for 5 s (U‐HGLGPS lamp). Scale bars: 100 µm.

Notably, photo‐responsiveness of A2Az and its effective adhesivity to M13 phage enabled site‐selective control of the virus infection in a three‐dimensional hydrogel environment (Figure 7a). We first performed live/dead assay for *E. coli* cells cultured in the A2Az hydrogel, indicating > 96% cell viability (Figure S34). Then, we prepared an A2Az (0.4 wt%) hydrogel in lysogeny broth (LB) medium containing M13 phage (1.3 × 10^11^ virions mL^−1^), *E. coli* cells, and SPiDER‐*β*Gal (0.6 µg mL^−1^). SPiDER‐*β*Gal is a substrate that upon cleavage by *β*‐galactosidase releases SPiDER, a dye that exhibits yellow fluorescence, indicating infection of *E. coli* cells by M13 phage.^[^ ^38^ ^]^ After the hydrogel sample was incubated for 7 days at 37 °C, fluorescence intensity of SPiDER hardly changed, as assessed by both visual observation (Figure 7b; *λ*_ex_ = 500 nm) and fluorescence spectroscopy (Figure 7d; blue and green, *λ*_ex_ = 500 nm). In contrast, after the hydrogel was irradiated with 350‐nm light for 30 s followed by incubation for 7 days at 37 °C, we observed increased fluorescence intensity compared to the non‐irradiated hydrogel (Figures 7c,d and S35). When we performed the plaque assay on each sample (*trans*‐A2Az before incubation, *trans*‐A2Az, and *cis*‐A2Az), the amounts of M13 phage were (2.7 ± 0.6) × 10^10^, (2.9 ± 0.7) × 10^10^, and (3.9 ± 0.5) × 10^12^ virions mL^−1^, respectively ((mean ± SD), Figure 7e), consistent with the results of fluorescence observation. Given that an A4Az hydrogel containing M13 phage, *E. coli* cells, and SPiDER‐*β*Gal exhibited an increase in fluorescence intensity due to SPiDER dye after 7‐day incubation even without light irradiation, the strong adhesive property of A2Az to M13 phage is likely necessary for effective inhibition of infection (Figure S36). Taking advantage of the high manipulability of photo irradiation, site‐selective control of M13 phage infection was attempted in a three‐dimensional environment. We prepared A2Az hydrogel containing M13 phage, *E. coli* cells, and SPiDER‐*β*Gal on a glass substrate, and a selective part of the hydrogel was irradiated with UV light (*λ* = 370 nm) under an optical microscope. After incubation of the hydrogel for 3 days at 37 °C, microscopic observation revealed distinct phase contrast images between the nonirradiated and light‐irradiated regions (Figure 7f). Importantly, fluorescence due to SPiDER dye was observed only in the light‐irradiated region, as visualized by 3D CLSM (Figure 7g). We also confirmed that M13 phage maintained the infection activity after the UV irradiation in this condition (Figure S37), and reconstruction of A2Az fiber network again inhibited the M13 phage infection (Figure S38). Thus, we successfully demonstrated site‐selective control of M13 phage infection with *E. coli* in a 3D hydrogel environment by the light‐triggered depolymerization of A2Az.

## Conclusion

Here, we reported on the photoresponsive hybrid of M13 phage and an amphiphilic peptide, A2Az, bearing an Az group near its N‐terminus. Thanks to the characteristic molecular design to attach the bulky aromatic group at the side chain of the amphiphilic peptide, A2Az self‐assembles into helical supramolecular fibers and exhibits effective interaction with M13 phage for the hybridization. The M13 phage and A2Az hybrid undergoes disassembly by photo‐induced depolymerization of A2Az fibers, enabling site‐selective control of not only the adhesion of M13 phage on 2D substrates but also the infection event in 3D hydrogel environments. Because of its characteristic anisotropic shape and surface adjustability, M13 phage can be utilized for the development of surfaces with electronic device functionality. Furthermore, designing M13 phages that can bind to inorganic substances such as nanoparticles is also possible. Therefore, the multidimensional photo‐patterning of M13 phage demonstrated in this work should contribute to advancing and expanding such applications of functional surface development and immobilization of optical functional nanoparticles. Given that A2Az fibers worked as scaffolds for cell culture (Figures S39) and M13 phage can be utilized as a gene vector for mammalian^[^ ^39^ ^]^ as well as *E. coli* cells, we envision that the hybridization of M13 phage and a helix‐forming self‐assembling peptide hydrogel with a visible light‐responsive aromatic side group likely provides a novel material design for a cell scaffold, permitting three‐dimensional photo‐manipulation of cells.^[^ ^40^, ^41^, ^42^ ^]^

## Supporting Information

The complete description of the materials, methods, and additional data is provided in Supporting Information.

## Conflict of Interests

The authors declare no conflict of interest.

## Supporting information



Supporting Information

## Data Availability

The data that support the findings of this study are available in the Supporting Information of this article.
