# Supplementary material for: A Photoresponsive Hybrid of Viruses and Supramolecular Peptide Fibers for Multidimensional Control of Patterning and Infection
Source: Angew Chem Int Ed Engl. 2025 Aug 25;64(41):e202508528. doi: 10.1002/anie.202508528 (PMC12501715; doi:10.1002/anie.202508528)
Supplement: Supplementary file 1 — Supporting Information [file ANIE-64-e202508528-s001.pdf]

Supporting Information

A Photoresponsive Hybrid of Viruses and Supramolecular Peptide Fibers for Multi-dimensional Control of Patterning and Infection

Atsuya Yaguchi, Noriyuki Uchida*, Daiki Miura, Go Watanabe, Hirotsugu Hiramatsu, Itsuki Ajioka, Teruhiko Matsubara, Toshinori Sato, Chinbat Enkhzaya, Shunto Itani, Tomokazu Saito, Takahiro Muraoka*

1. Materials

Acetic anhydride was purchased from Kanto Chemicals (Tokyo, Japan). Acetonitrile, *N*,*N'*-dimethylformamide (DMF), Et_2O, *N*-methyl-2-pyrrolidone (NMP), piperidine, and trifluoroacetic acid (TFA) were purchased from Kishida Chemical (Tokyo, Japan). *N*,*N*-Diisopropylethylamine (DIEA), polyethylene glycol (PEG), and tryptone were purchased from Nacalai Tesque (Kyoto, Japan). 4',6-Diamidino-2-phenylindole (DAPI), gold nanoparticles (400 nm diameter), anti-fd bacteriophage antibody, and fibronectin were purchased from Sigma (St. Louis, MO, U.S.A.). Dulbecco's modified eagle medium (DMEM, high glucose), fetal bovine serum (FBS), LIVE/DEAD™ *Bac*Light™ Bacterial Viability and Counting Kit, and penicillin-streptomycin were purchased from Thermo Fisher Scientific (Waltham, MA, U.S.A). TNBS Test Kit, triisopropylsilane (TIS), and tetracycline hydrocholoride were purchased from Tokyo Chemical Industry (Tokyo, Japan). Fmoc-Ala-OH·H_2O, Fmoc-Arg(Pbf)-OH, Fmoc-Asp(OtBu)-OH, Fmoc-NH-SAL Resin, Fmoc-Phe(4-N=NPh)-OH, 2-(1*H*-benzotriazole-1-yl)-1,1,3,3-tetramethyluronium hexafluoro- phosphate (HBTU), and 1-hydrozybenzotriazole monohydrate (HOBt·H_2O) were purchased from Watanabe Chemical Industries (Hiroshima, Japan). Ultrapure water (filtered through a 0.22 μm membrane filter, >18.2 MΩ cm) was purified in Purelab DV35 of ELGA (Buckinghamshire, UK). *E. coli* ER2738 and M13KE bacteriophage (M13 phage) were purchased from New England Biolabs (Ipswich, MA, USA). SPiDER-*β*Gal was purchased from DOJINDO (Kumamoto, Japan). Rhodamine B isothiosianate and goat anti-rabbit IgG antibody (HRP conjugate) were purchased from Cosmo Bio (Tokyo, Japan). Agar powder, 5-bromo-4-chloro-3-indolyl *β*-D-galactopyranoside (X-gal), agarose S, isopropyl *β*-D-thiogalactoside (IPTG), tetracycline hydrochloride, yeast extract, bovine serum albumin (BSA), phosphate buffered saline (PBS), and gelatin were purchased from FUJIFILM Wako Chemicals (Osaka, Japan). 13 mm-*φ* cover glass was purchased from Matsunami Glass Ind., Ltd. (Osaka, Japan).

2. Instruments

Matrix-assisted laser desorption/ionization time-of-flight (MALDI-TOF MS) was performed on autoflex speed spectrometer of Bruker (Bremen, Germany) in a reflector positive mode with 2,5-dihydroxybenzoic acid as a matrix. Circular dichroism (CD) spectra were recorded on J-1100 CD spectrometer of JASCO (Tokyo, Japan) with PTC-514 peltier temperature controller. Light irradiation was performed with MAX-303 300 W Xe-lamp of Asahi Spectra (Tokyo, Japan) through a filter (λ = 350 ± 5 nm, λ = 450 ± 5 nm, and λ = 500 ± 5 nm) or U-HGLGPS (130 W) of Olympus (Tokyo, Japan) through a U-FUNA filter (λ = 365 ± 5 nm). Infrared spectra (IR) were recorded on FT/IR-6100 of JASCO (Tokyo, Japan). UV-Vis absorption spectra and diffuse reflectance spectra were recorded on V770 spectrometer of JASCO (Tokyo, Japan). Fluorescence spectra were measured on RF-6000 spectrofluorometer of Shimadzu (Kyoto, Japan). Rheological analyses were conducted by Kinexus rotational rheometer of Malvern Panalytical (Malvern, UK) Transmission electron microscopic (TEM) observations were conducted by JEM-1400 of JEOL (Tokyo, Japan) equipped with Gatan UltraScan 4k×4k CCD camera using JEOL carbon reinforced microgrid (Cu 200 mesh) and uranyl acetate or gadolinium acetate for staining under 200 kV accelerating voltage, and a photographing method of JEOL minimum dose system was used (MDS, search/focus/record modes). Peptide syntheses were conducted with HiPep Laboratories model shaking apparatus of HiPep Laboratories (Kyoto, Japan) using polypropylene LibraTubes with a filter. Sonication was conducted with ultrasonic bath sonicator AS12GTU (35 kHz, 60 W) of As One (Osaka, Japan). Lyophilization was conducted by an FDU-1200 lyophilizer of EYELA (Tokyo, Japan). Centrifugation was conducted by Kubota type micro refrigerated centrifuge 3700. Incubation for the hydrogel preparation was conducted by a peltier cool incubator CN-40A of Mitsubishi Electric Engineering (Tokyo, Japan). Fluorescence microscopic observations were conducted by IX73 microscope of Olympus (Tokyo, Japan) attached with a Hamamatsu Photonics model CCD camera C10600-10B. Powder X-ray diffraction (PXRD) measurements were carried out by Nano-Viewer system of Rigaku (Tokyo, Japan) equipped with Dectris PILATUS 100k detector ($Cu_{K\alpha}$). Scanning electron microscopy (SEM) was performed by a S-4500 of Hitachi (Tokyo, Japan). Confocal laser scanning microscopy (CLSM) was performed by LSM 900 of Zeiss (Jena, Germany) or STELLARIS 5 of Leica (Wetzlar, Germany). Zeta potential measurements were performed by Panalytical Zetasizer Nano ZSP of Malvern Panalytical (Malvern, U.K.). Ion

conductivity was measured by DS-72E of Horiba (Kyoto, Japan). Differential scanning calorimetry (DSC) was performed by DSC7000X of Hitachi (Tokyo, Japan) using an aluminum pan GCA-0017 of Hitachi High-Tech (Tokyo, Japan).

3. Peptide synthesis

Ac-RAzDARADA-NH_2 (A2Az), Ac-RADAzRADA-NH_2 (A4Az), Ac-RADARAzDA-NH_2 (A6Az), Ac-RADARADA-NH_2 (RADA8), Ac-RAzDA-NH_2 (RAzDA), Ac-$(RADA)_4$-NH_2 (RADA16), Ac-RNpDARADA-NH_2 (A2Np), Ac-RADNpRADA-NH_2 (A4Np), Ac-RBpDARADA-NH_2 (A2Bp), and Ac-RADBpRADA-NH_2 (A4Bp) were synthesized by 9-fluorenylmethyloxycarbonyl (Fmoc) solid-phase peptide synthesis according to that reported previously.[17,18] Np and Bp represent naphthyl- and biphenyl-containing amino acid residues, respectively. Typically, a condensation-reagents cocktail of HBTU (3.1 g, 8.0 mmol) and HOBt·H_2O (1.3 g, 8.2 mmol) in DMF (16 mL), a mixture of DIEA (2.8 mL) and NMP (14.3 mL), and a cleavage cocktail of TIS (62.5 μL), TFA (2.4 mL), and water (62.5 μL) were prepared just prior to the synthesis. Fmoc-NH-SAL Resin (0.1 mmol) in a polypropylene tube was soaked in DMF (2 mL) over 3 h at 25 °C. After removal of DMF, piperidine in DMF (20%, 2 mL) was added and mixed with a vortex device for 1 min. After the reaction solution was removed, piperidine in DMF (20%, 2 mL) was added and the reaction tube was shaken for 10 min at 25 °C. After removal of the reaction solution, the resin was washed with DMF (2 mL, 5 times), CH_2Cl_2 (2 mL, 3 times) and DMF (2 mL, 3 times). To the resin was added Fmoc-protected amino acid (0.3 mmol) dissolved in the condensation-reagents cocktail (700 μL) and the mixture of DIEA and NMP (700 μL). After shaking for 15 min at 25 °C, the reaction solution was removed and the resin was washed with DMF (2 mL, 5 times), CH_2Cl_2 (2 mL, 3 times) and DMF (2 mL, 3 times). The Fmoc deprotection reactions with piperidine and coupling reactions of Fmoc-protected amino acid were repeated following the designed sequence. After the final Fmoc deprotection reaction and washing, acetic anhydride in CH_2Cl_2 (25%, 2 mL) was added to the resin, and the reaction tube was shaken for 5 min at 25 °C. After removal of the reaction solution, the resin was washed with DMF (2 mL, 5 times), CH_2Cl_2 (2 mL, 3 times) and DMF (2 mL, 3 times). The cleavage cocktail (2.5 mL) was added to the resin, and the reaction tube was left to stand for 90 min at 25 °C with gentle shaking every 30 min. The solution was collected into a polypropylene centrifuge tube by filtration. The reaction tube was rinsed with TFA (500 μL, 3 times), which is also collected by filtration. To the centrifuge tube was added Et_2O (40 mL) and the tube was mixed on a vortex device for 1 min and centrifuged at 4 °C (3500 × g, 5 min) followed by removal of the supernatant liquid. After repeating

this process for 3 times, the peptide was dried under vacuum over 2 h at 25 °C, dispersed in water and lyophilized.

MALDI-TOF MS (2,5-dihydroxybenzoic acid, reflector positive): calculated monoisotopic masses: 653.31 for RAzDA + H^+ ($C_{30}H_{41}N_{10}O_7^+$), 1066.51 for A2Az + H^+ ($C_{46}H_{68}N_{17}O_{13}^+$), A4Az + H^+ ($C_{46}H_{68}N_{17}O_{13}^+$), A6Az + H^+ ($C_{46}H_{68}N_{17}O_{13}^+$), 886.45 for RADA8 + H^+ ($C_{34}H_{60}N_{15}O_{13}^+$), 1012.50 for A2Np + H^+ ($C_{44}H_{66}N_{15}O_{13}^+$), 1012.50 for A4Np + H^+ ($C_{44}H_{66}N_{15}O_{13}^+$), 1038.51 for A2Bp + H^+ ($C_{46}H_{68}N_{15}O_{13}^+$), and 1038.51 for A4Bp + H^+ ($C_{46}H_{68}N_{15}O_{13}^+$); observed *m*/*z*: 653.35 (RAzDA), 1066.76 (A2Az), 1066.65 (A4Az), 1066.74 (A6Az), 886.437 (RADA8), 1012.54 (A2Np), 1012.68 (A4Np), 1038.57 (A2Bp), 1038.69 (A4Bp).

4. Methods

Self-assemblies of peptides

Typically, a lyophilized powder of A2Az (14.3 mg) was dissolved in 1.43 mL of TFA solution (2.2 v/v%). After gentle shaking and sonication at 25 °C, the sample was allowed to stand overnight at 20 °C. Self-assemblies of A4Az, A6Az, RADA8, RADA16, RAzDA, and A2Az/RADA8 (A2Az:RADA8, weight ratio 80:20) were prepared using the same method except that A2Az (14.3 mg) was replaced with A4Az (14.3 mg), A6Az (14.3 mg), RADA8 (11.4 mg), RADA16 (14.3 mg), RAzDA (8.6 mg), or a mixture of A2Az (11.4 mg) and RADA8 (2.9 mg). For embedding M13 phage and *E. coli* cells, A2Az (0.5 wt%) and A4Az (0.5 wt%) fiber hydrogels were prepared by the same method but using phosphate-buffered saline (pH 7.4) instead of TFA solution.

Computational details

All-atom molecular dynamics (MD) simulations were performed using the GROMACS software package (Version 2020.5).[19] For each system, 56 peptide molecules were randomly inserted into the initial cubic simulation box with sides of length 10 nm, based on the previous study.[15] The rest of the space was filled with approximately 30,000 water molecules. Successive 2-ns relaxation runs at 250 K and 310 K under the periodic boundary conditions were conducted following the steepest descent energy minimization. After the relaxation runs, the equilibration MD run was performed at 1 bar and 310 K for 300 ns under the periodic boundary conditions for each system. The Amber ff99SB-ILDN force field[20] was used for the force field parameters of the peptides, the generalized Amber force field was used for the Az, and the TIP4P-EW model was used for the water molecules. During the relaxation runs, the carbon, nitrogen, and oxygen atoms of the peptides were constrained to their initial positions with a force constant of 1,000 $kJ\cdot mol^{-1}\cdot nm^{-2}$. The velocity-rescaling[21] and Berendsen barostat[22] were used to maintain the temperature and pressure of the system, employing relaxation times of 0.2 and 2.0 ps, respectively. The equilibration runs were performed using the Nosé-Hoover thermostat[23–25] and Parrinello-Rahman barostat,[26] employing relaxation times of 1.0 and 5.0 ps, respectively. The all-bonds connected to hydrogen atoms were constrained with the LINear Constraint Solver (LINCS) algorithm;[27] the time step was set to 2 fs. The smooth particle-mesh Ewald method[28] was used to calculate the long-range Coulomb interactions. The real space cutoff and the grid spacing were 1.4 and 0.30 nm,

respectively. The force field parameters related to the C-N=N-C dihedral angle of the Az moiety were modified using the proper dihedral angle potential function, as follows:

$$V_d(\phi_{ijkl}) = 12.129 \cdot (1 + \cos\phi) + 51.927 \cdot (1 + \cos(2\phi - 180))$$

IR analysis of self-assemblies of peptides

IR spectra of self-assemblies of A2Az, A4Az, and A6Az were measured in the hydrated state using CaF_2 windows, for which the path length was fixed (Biocell; Biotools, FL). Band decomposition analysis of the IR spectra was conducted using six Gaussian bands, as reported previously.[17] Since the amide I bands of α-helical and random coil overlap at a wavenumber region higher than 1640 cm^{-1}, the bands at wavenumber regions lower and higher than 1640 cm^{-1} were assignable to β-sheet structure (lower) and random coil or α-helix (higher). For A2Az, A4Az, A2Az/RADA8 (A2Az:RADA8, weight ratio 80:20), and RADA16, the ratios between hydration-derived and the amide-derived peaks were evaluated by integrating the intensities of the IR signals from 2600 to 3800 cm^{-1} and from 1500 to 1800 cm^{-1}, respectively. Signals related to TFA were eliminated by the background measurement of TFA in water. When residual peaks of TFA were observed, they were removed by using the separately measured spectrum of TFA in water. The TFA peaks at 1150 and 1200 cm^{-1}, and accordingly, that at 1670−1675 cm^{-1}, were eliminated.

Contact angle measurement

A glass plate (Matsunami Micro Cover Glass, 18×18mm, No.1) was immersed into an aqueous solution of NaOH (0.1 M) for 15 min at 25 °C followed by washing with deionized water and air drying. A hydrogel of a peptide (1.0 wt%, 150 µL) was placed on the glass plate. After air-drying, the peptide-coated glass was rinsed with deionized water once, which was then dried by N_2 flow over 5 min at 25 °C. A drop of deionized water (1.0 µL) was placed onto the peptide-coated glass using a glass syringe with a 22-gauge stainless needle and the photograph was taken by a CCD camera. The contact angle of the water drop was analyzed by a software of Kyowa Interface Science, where the contact angle was calculated based on a half-angle (θ/2) method.

DSC analysis of self-assemblies of peptides

Hydrogels containing 1.0 wt% of A2Az (5.8 mg), A4Az (7.9 mg), a mixture of A2Az and RADA8 (A2Az/RADA8, weight ratio 80:20) (11.27 mg in total), and RADA16 (19.3 mg) were prepared for measurement. The samples were heated from −80 to 50 °C at a rate of 2 °C min^{-1}, and ΔH values were calculated by integrating the peaks from −10 to 15 °C.

Handling of M13 phage

M13 phage was amplified using *E. coli* cells in LB medium containing tetracycline (45 μM) and purified by two cycles of precipitation and re-dispersion using a solution containing PEG (M_w = 8000, 14 wt%) and NaCl (290 mM) according to a previously reported method.[29,30] The density of M13 phage was quantified by performing plaque assays on X-gal plates.[31] For the preparation of Cy5-labeled M13 phage, a suspension of M13 phage (8.9 × 10^{13} virions/mL) in Tris-HCl buffer (1 mM Tris-HCl, pH 7.0) was incubated with Cyanine5−N-hydroxysuccinimide (Cy5-NHS) ester (2.5 mg/mL) and incubated for 6 h. The sample then was centrifuged (286,000 *g*) for 60 min, and the pellet was re-dispersed in water.[32] For use in the experiment with M13 phage, *E. coli* cells (ER2738) were prepared by inoculating LB medium containing tetracycline with a bacterial colony of *E. coli* (obtained from LB agar) and incubating overnight at 37 °C. For evaluating binding affinity of M13 phage onto the substrate, was utilized according to reported method.[33] Gold-binding M13 phage (M13 phage^{Au}) was prepared by genetically introducing a peptide with a gold-binding ability (VSGSSPD)[34] via a GGGS linker to the N-terminus of the P_{III} coat protein of M13-Non according to the procedure in the literature.[35]

M13 phage adhesion assay

As a typical procedure for investigating the adhesion of M13 phage to substrates, a hydrogel of A2Az (0.7 wt%, 200 μL) was placed on a glass coverslip. After air-drying of the hydrogel, the A2Az fiber-coated coverslip was rinsed twice with water and once with Tris-HCl buffer (1 M Tris-HCl, pH 7.5). An aliquot (100 μL) of an aqueous suspension of M13 phage (17.9 × 10^{11} virions/mL) was dropped on the A2Az fiber-coated substrate and incubated for 90 min at 25 °C; the substrate then was rinsed twice with the Tris-HCl buffer and twice with water. Then, M13 phage that had adhered to the substrate were eluted with glycine buffer (200 mM glycine, pH 2.2), and the eluate was neutralized with a Tris-HCl buffer (1 M Tris-HCl, pH 8.5) prior to use in the plaque assay.

The same method was employed for M13 phage adhesion assays for a variety of peptide-coated coverslips. For the deposition of gold nanoparticles onto the substrate, M13 phage (2.8×10^{-14} virions/mL) or M13 phage^{Au} (2.8×10^{-14} virions/mL) was incubated with gold nanoparticles in Tris-HCl buffer (50 mM Tris-HCl, pH 7.4) for 60 min at 25 °C. After centrifugation (10000 × rpm) of the sample at 25 °C for 2 min, the precipitate was re-dispersed in Tris-HCl buffer (50 mM Tris-HCl, pH 7.4) and incubated with an A2Az fiber-coated substrate.

As a typical procedure for enzyme-linked immuno-sorbent assay (ELISA) assay, to fiber-coated substrates, 200 µL of PBS/1% BSA was injected and let stand at room temperature for 2 h. After washing three times with PBS, 50 µL of M13 phage solutions were added and incubated at room temperature for 2 h. After washing the substrates five times with PBS, the samples were incubated with 100 µL of PBS solution containing BSA (1 wt%) and 1/1000 diluted anti-fd bacteriophage antibody (code B-7786, Aldrich) at room temperature for 1 h. After washing the substrates five times with PBS, the samples were incubated with 100 µL of PBS solution containing BSA (1 wt%) and1/1000 diluted anti-rabbit IgG peroxidase conjugate antibody (code A-8275, Aldrich) at room temperature for 1 h. After five times washing with PBS, the samples were incubated with 100 µL of a citric acid phosphate buffer solution (pH 5.0) containing H_2O_2 (0.02% v/v) and o-phenylenediamine (0.4 mg/mL) at room temperature for 15 min. Then, 100 µL of 3 N H_2SO_4 was added to quench the reactions, and the absorption at 492 nm was measured using a microplate reader.

Photo-selective detachment of M13 phage from A2Az fiber-coated substrate

An aliquot (100 µL) of an aqueous suspension of Cy5-labeled M13 phage (17.9×10^{11} virions/mL) was dropped on a peptide-coated substrate and incubated for 90 min. The substrate then was rinsed twice with a Tris-HCl buffer (1 M Tris-HCl, pH 7.5) and twice with water. Next, the substrates were partially covered with aluminum foil as a photomask and irradiated with 350-nm light for 40 min. The substrate was washed twice with a Tris-HCl buffer (1 M Tris-HCl, pH 7.5) and twice with water prior to observation by CLSM.

Photo-induced infection by M13 phage of *E. coli* cells in peptide hydrogels

For the photo-manipulation of M13 phage infection, an aliquot (100 μL) of A2Az (0.5 wt%) or A4Az (0.5 wt%) hydrogel was incubated for 3 min with 30 μL of an aqueous suspension of M13 phage (5.8×10^{11} virions/mL), followed by combining with 12.5 μL of a 100-fold amplified *E. coli* cell sample supplemented with SPiDER dye (6.0 μg/mL), and isopropyl *β*-D-thiogalactopyranoside (2.2 mM); the resulting mixture was incubated for 7 days at 37 °C. To investigate photo-responsiveness, the hydrogel sample was irradiated with 350-nm light before the incubation. An assay of the site-selective induction of M13 phage infection was performed by the same method except that the mixture was incubated for 3 days instead of 7 days.

Light irradiation

350- and 450-nm light irradiations of A2Az fiber and A4Az fibril samples in the test tube or A2Az hydrogel film on the glass substrate were performed using a MAX-303 Xe lamp (300 W, 0.5 mW/cm^{-2}, Asahi Spectra, Tokyo, Japan) equipped with appropriate band-pass filters ($\lambda = 350 \pm 5$ nm and 450 ± 5 nm). The 350-nm irradiation (in test tubes) of A2Az and A4Az hydrogels containing M13 phage and *E. coli* cells was performed using the Xe lamp for 30 s. The irradiation of A2Az hydrogel containing M13 phage and *E. coli* cells coated onto a glass substrate was performed for 5 s using a U-HGLGPS lamp (130 W, 8.97 mW/cm^{-2}, Olympus, Tokyo, Japan) equipped with a U-FUNA filter ($\lambda = 365 \pm 5$ nm) on an IX-73 microscope (Olympus, Tokyo, Japan). The light exposure time was adjusted depending on the power of the light source and the thickness of the samples.

Adhesion of lysozyme to peptide-coated substrates

For the preparation of the peptide-coated substrate for this assay, a dispersion of A2Az (1.0 wt%) in TFA solution (2.2 v/v%) was dried on a glass substrate and washed twice with water. Substrates coated with A4Az (1.0 wt%), RADA16 (1.0 wt%), or a mixture of A2Az and RADA8 (A2Az:RADA8 = weight ratio 80:20) (1.0 wt%) were prepared by the same method. Fluorescent rhodamine B-labeled lysozyme (lysozyme^{Rho}) was prepared by mixing lysozyme (20.7 mg/mL) with rhodamine B isothiocyanate (4.7 mg/mL) in Tris-HCl buffer (50 mM Tris-HCl, 300 mM NaCl, pH 7.5) and incubating overnight at 25 °C, followed by purification by high-performance liquid chromatography (HPLC). To evaluate the adhesion of lysozyme^{Rho}, an aqueous solution of

lysozyme^{Rho} (1.6 mg/mL) was incubated with the peptide-coated substrates for 1 min at 25 °C. After removal of the solution containing non-adhering lysozyme^{Rho}, the amount of lysozyme^{Rho} adhering to the substrate was evaluated by fluorescence spectroscopy (λ_{ex} = 550 nm, λ_{obs} = 580 nm).

Cell adhesion assay

As a typical procedure for investigating adhesion of NIH 3T3 fibroblasts (fibroblasts) on substrates,[36] a hydrogel of A2Az fiber (0.7 wt%, 20 μL) was placed on a 13 mm-φ cover glass. After air-drying, the A2Az fiber-coated cover-glass was rinsed with phosphate-buffered saline (PBS). Fibroblasts (3.6×10^5 cells) were suspended in Dulbecco's Modified Eagle Medium including fetal bovine serum (10%) and penicillin-streptomycin, plated on the A2Az fiber-coated cover glasses, and incubated in a CO_2 (5%) incubator. After 3 h, the cells were gently washed with PBS and fixed with paraformaldehyde (4%) for 15 min at 25 °C. The fixed cells were washed with PBS including 0.5% Triton X-100 three times and stained with DAPI (2.0 μg/mL) for 10 min. Fluorescence micrographs were captured using a fluorescence microscope. The DAPI-positive nuclei were counted by unbiased 2D stereology (Stereo Investigator, MBF Bioscience, VT, U.S.A.). Adhesion assays for fibroblasts on fibronectin-coated glasses were performed in the same method using A4Az (0.5 wt%, 20 μL) and fibronectin (50 ng/μL, 20 μL), respectively. As a control, cell adhesion assay glass substrate without coating was also tested.

5. Supporting figures

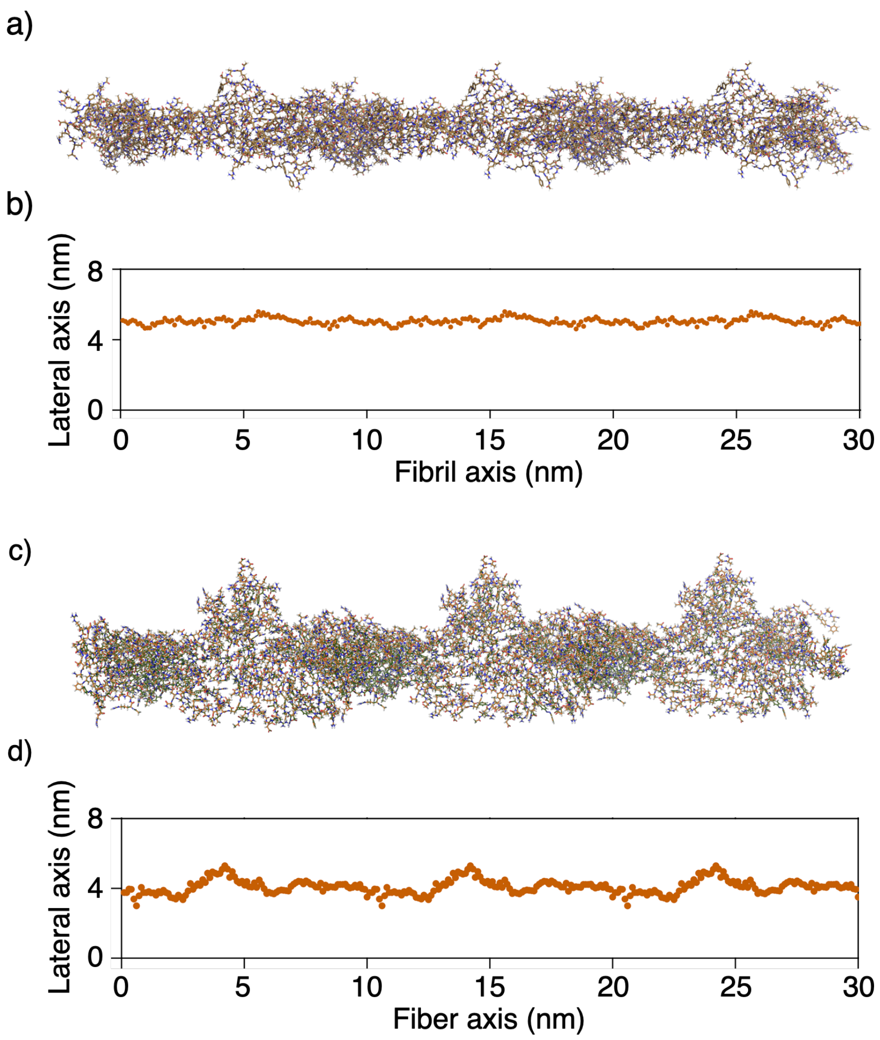

Figure S1. a, b) Snapshot of self-assembling structure (a) and its distribution of the particle center of mass (b) of A4Az in water at 300 K calculated by all-atom MD simulations. c, d) Snapshot of self-assembling structure (c) and its distribution of the particle center of mass (d) of A6Az in water at 300 K calculated by all-atom MD simulations.

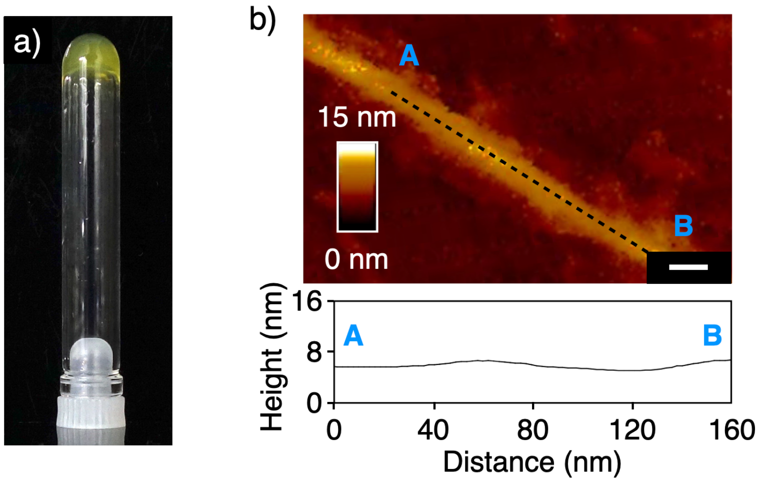

Figure S2. a) Photograph of hydrogel of the A4Az fibril (1.0 wt%) containing trifluoroacetic acid (TFA; 2.2 v/v%) at 20 °C. b) Atomic force microscopy (AFM) image with height profiles of the A4Az fibril. Scale bars: 20 nm. The dashed line indicates the region of the height profile.

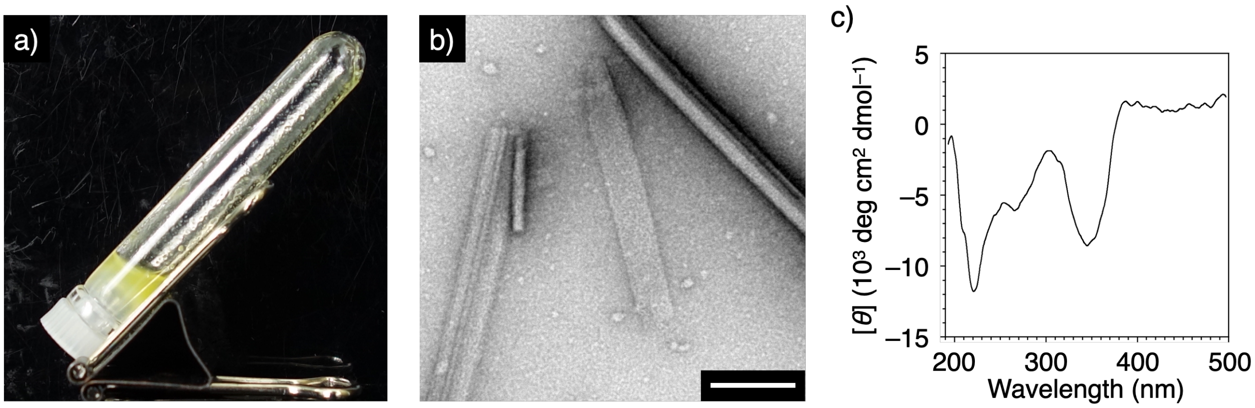

Figure S3. a) Photograph, b) TEM image, and c) CD spectrum of self-assembly of A6Az (1.0 wt%) in water containing TFA (2.2 v/v%). In (b), Scale bar: 100 nm, and the TEM sample was stained with uranyl acetate.

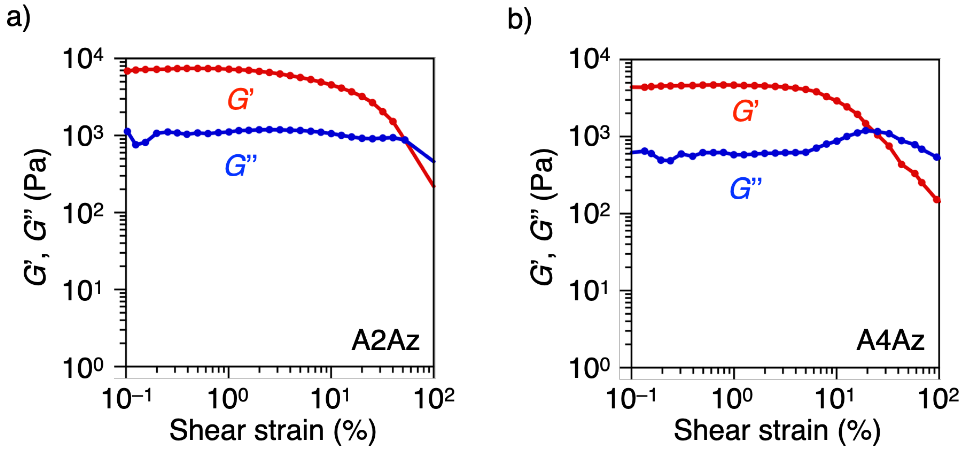

Figure S4. a, b) Strain-dependent storage moduli *G*' (red) and loss moduli *G*'' (blue) of hydrogels composed of A2Az (1.0 wt%) (a) and A4Az (1.0 wt%) (b) in water containing TFA (2.2 v/v%) at 20 °C.

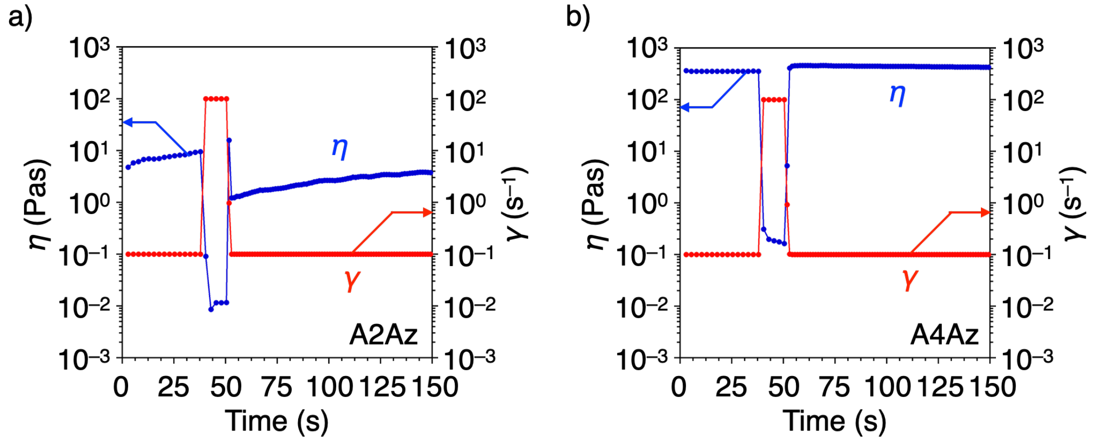

Figure S5. Flow viscosity (η) of hydrogels composed of a) A2Az (1.0 wt%) and b) A4Az (1.0 wt%) (b) in water containing TFA (2.2 v/v%) upon changing shear rate (γ) at 20 °C.

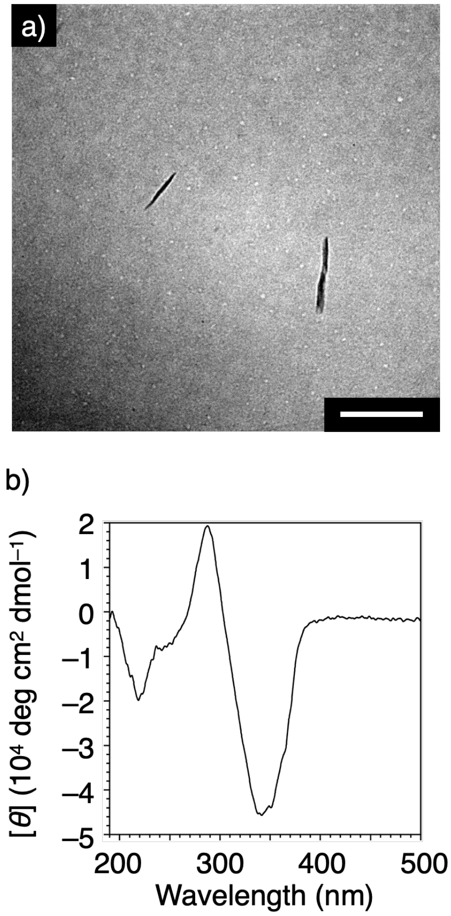

Figure S6. a) TEM image of RAzDA (0.6 wt%) in water containing TFA (2.2 v/v%). Scale bar: 500 nm. The TEM sample was stained with gadolinium acetate. b) CD spectrum of RAzDA (0.6 wt%) in water containing TFA (2.2 v/v%).

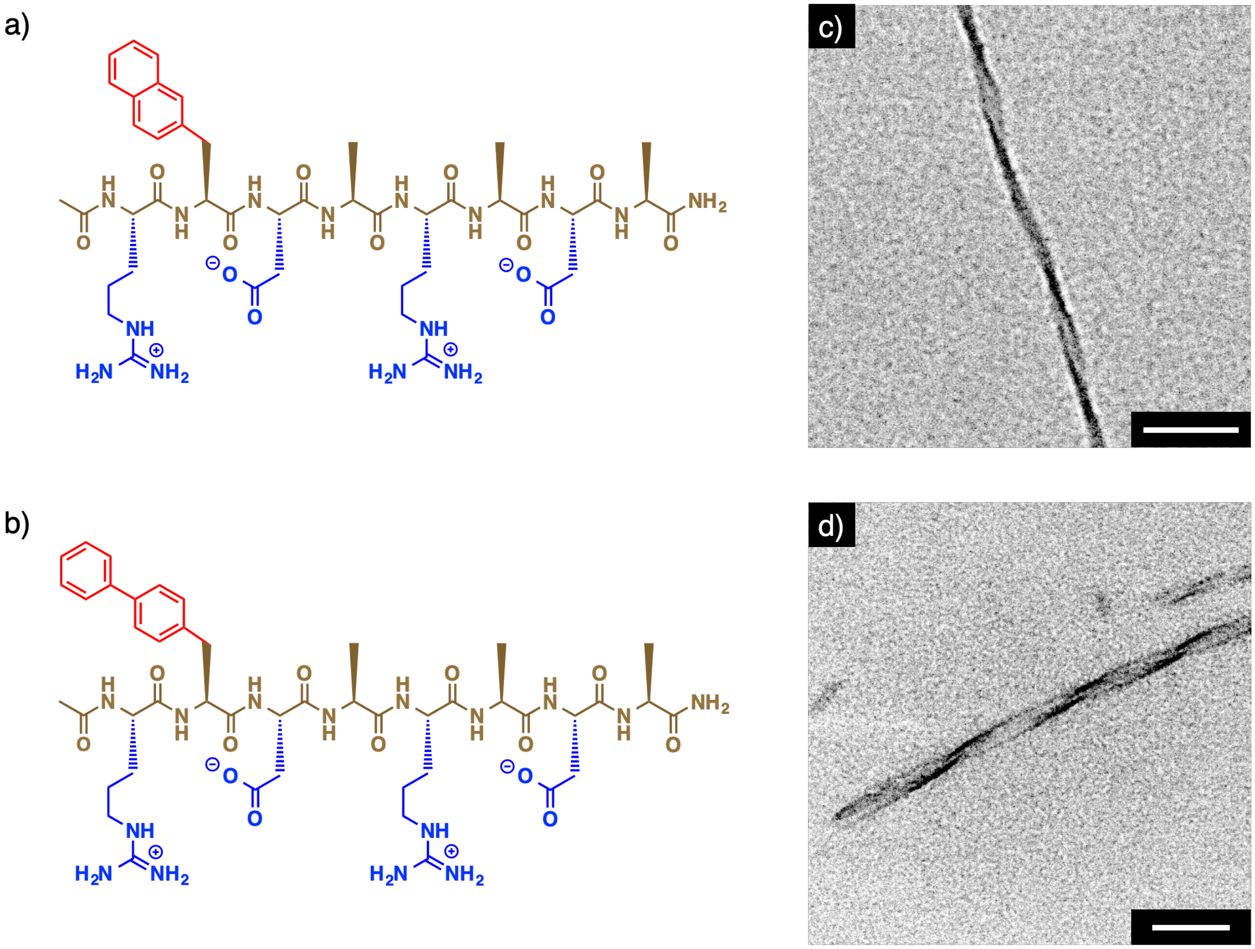

Figure S7. Molecular structures of a) naphthyl and b) biphenyl-introduced amphiphilic peptides instead of Az group of A2Az. TEM images of the fibers of c) naphthyl and d) biphenyl-introduced peptides (1.0 wt%) in water containing TFA (2.2 v/v%). Scale bars: 100 nm. The TEM samples were stained with uranyl acetate.

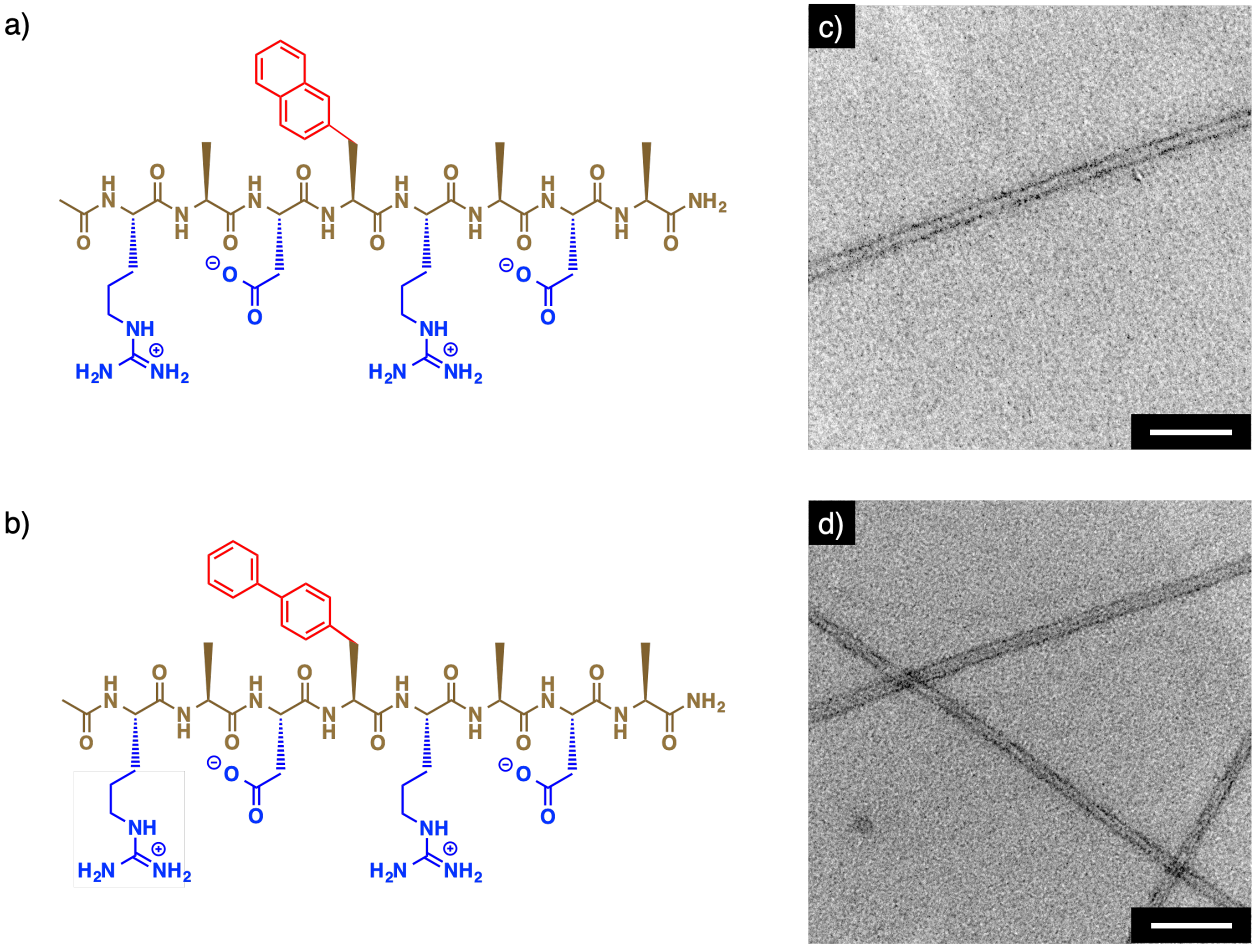

Figure S8. Molecular structures of a) naphthyl and b) biphenyl-introduced amphiphilic peptides instead of Az group of A4Az. TEM images of the fibers of c) naphthyl and d) biphenyl-introduced peptides (1.0 wt%) in water containing TFA (2.2 v/v%). Scale bars: 100 nm. The TEM samples were stained with uranyl acetate.

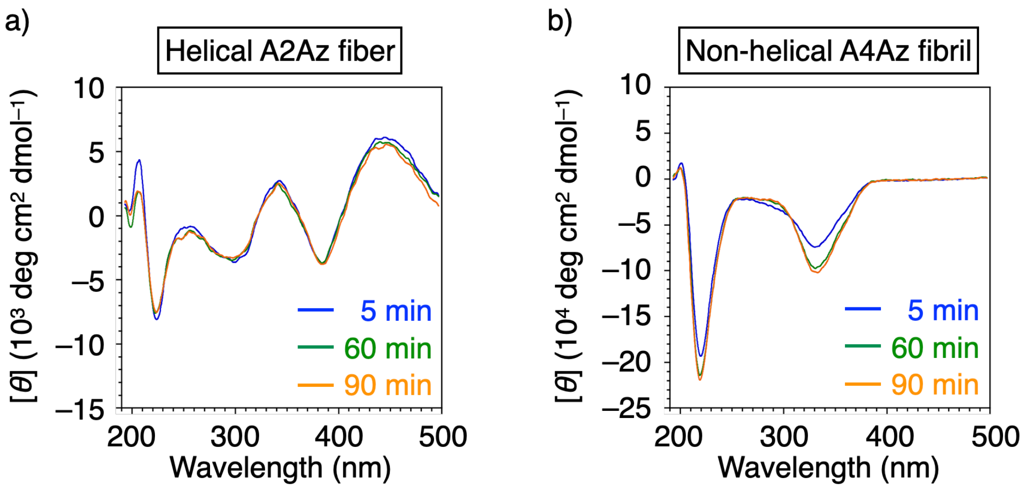

Figure S9. CD spectral changes of a) A2Az (1.0 wt%) and b) A4Az (1.0 wt%) in water containing TFA (2.2 v/v%) upon incubation for 5 min (blue), 60 min (green), and 90 min (orange) at 20 °C.

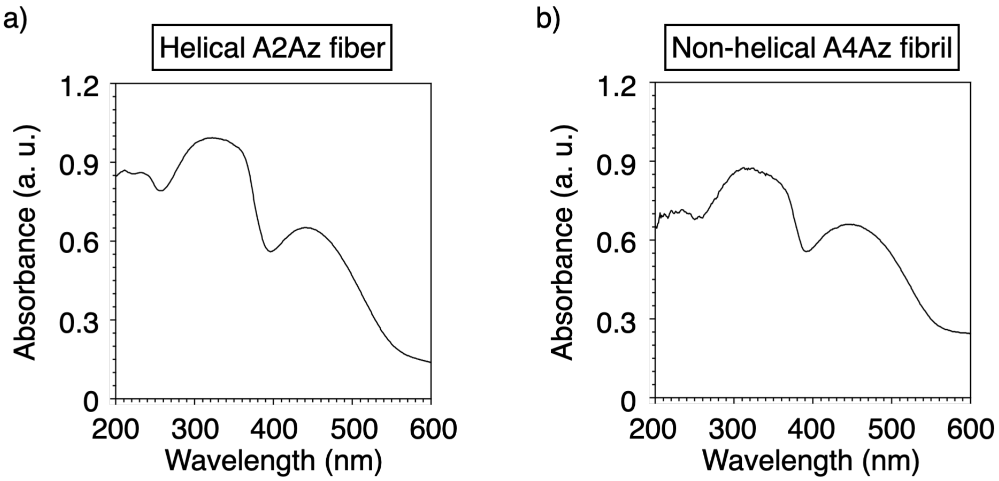

Figure S10. Diffuse reflectance spectra of the lyophilized hydrogels formed by a) A2Az (1.0 wt%) and b) A4Az (1.0 wt%).

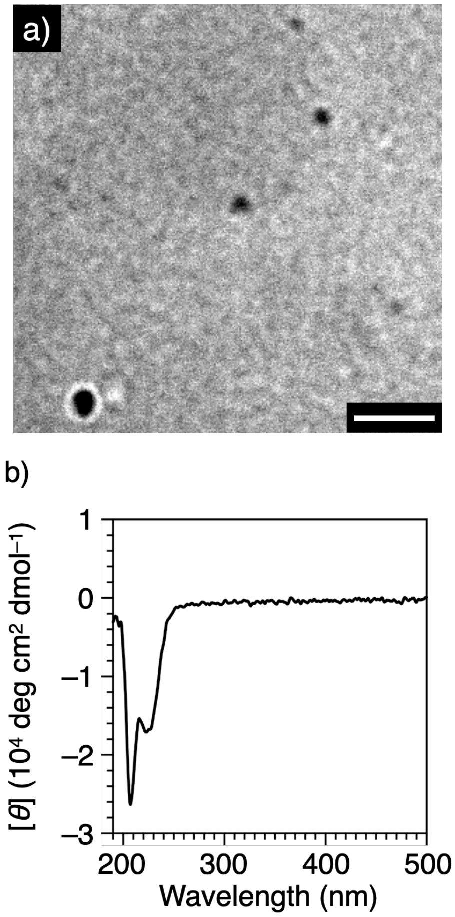

Figure S11. a) TEM image of RADA8 (0.8 wt%) in water containing TFA (2.2 v/v%). Scale bar: 500 nm. The TEM sample was stained with gadolinium acetate. b) CD spectrum of RADA8 (0.8 wt%) in water containing TFA (2.2 v/v%).

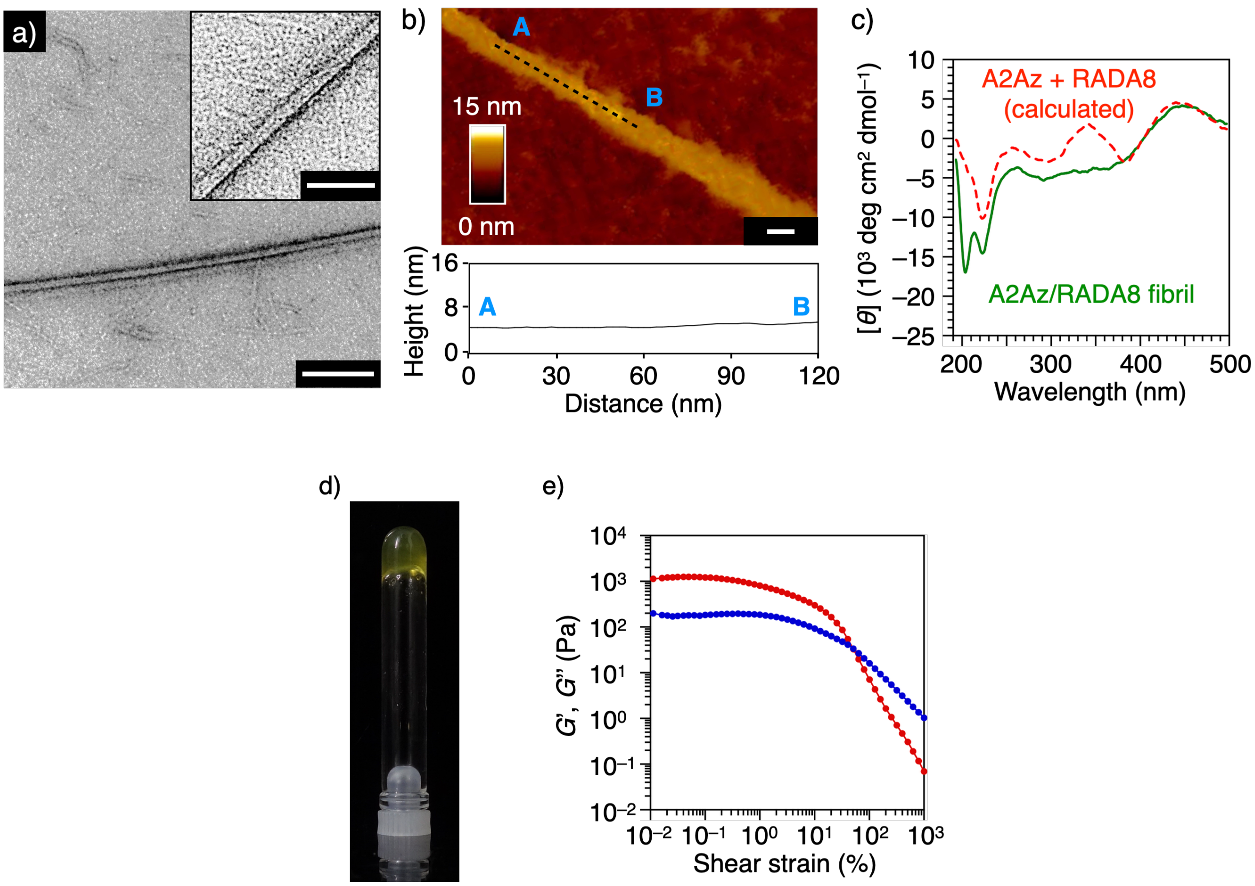

Figure S12. a) TEM with magnified image (inset) of a A2Az/RADA8 fibril (1.0 wt% in total) at the weight ratio of A2Az:RADA8 = 80:20. Scale bars: 100 nm and 50 nm (inset). The TEM sample was stained with uranyl acetate. b) AFM image with height profile of the A2Az/RADA8 fibril. Scale bar: 20 nm. The dashed line indicates the region of the height profile. c) CD spectra of the mixtures of A2Az and RADA8 (weight ratio of A2Az:RADA8 = 80:20, 1.0 wt% in total) in water containing TFA (2.2 v/v%), and simulated spectral profiles using the sum of individual CD spectra of A2Az and RADA8 (red dashed lines). d) Photograph and e) strain-dependent storage modulus *G*' (red) and loss modulus *G*'' (blue) of a hydrogel composed of the mixtures of A2Az and RADA8 (1.0 wt% in total) at the weight ratio of A2Az:RADA8 = 80:20 in water containing TFA (2.2 v/v%) at 20 °C.

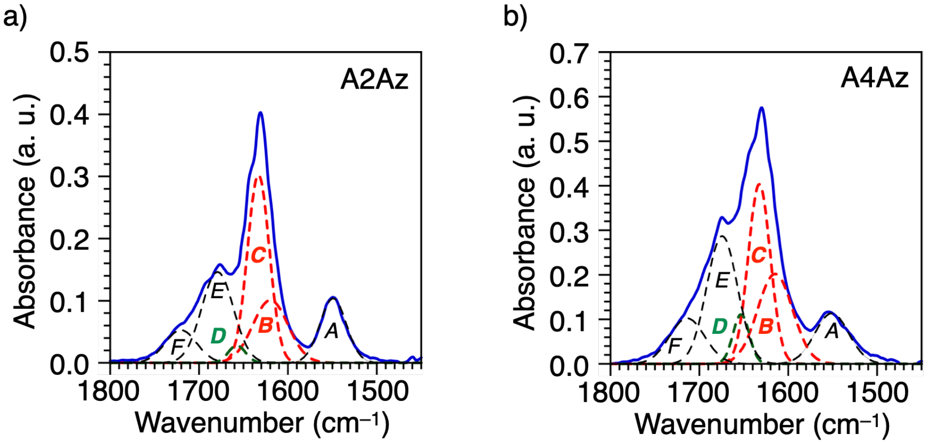

Detected bands (cm^{-1})	Detected bands (cm^{-1})
A: 1550 (Amide-II)	*A*: 1552 (Amide-II)
B: 1620 (β-Sheet)	*B*: 1615 (β-Sheet)
C: 1633 (β-Sheet)	*C*: 1632 (β-Sheet)
D: 1658 (Random coil or α-helix)	*D*: 1653 (Random coil or α-helix)
E: 1680 (Arginine)	*E*: 1675 (Arginine)
F: 1720 (COOH)	*F*: 1713 (COOH)

Figure S13. IR spectra (blue lines) of a) A2Az (1.0 wt%) and b) A4Az (1.0 wt%). Peaks assigned as A, B, C, D, E, and F by decomposition analysis correspond to amide-II, β-sheet, β-sheet, random coil or α-helix, arginine, and COOH, respectively.[15] Peaks corresponding to β-sheet (red dashed lines) are dominant in the amide-I bands (B, C, and D) in both samples and their percentages of β-sheet were 96% in A2Az fiber and 90% in A4Az fibril, respectively, as evaluated by the IR signal areas.

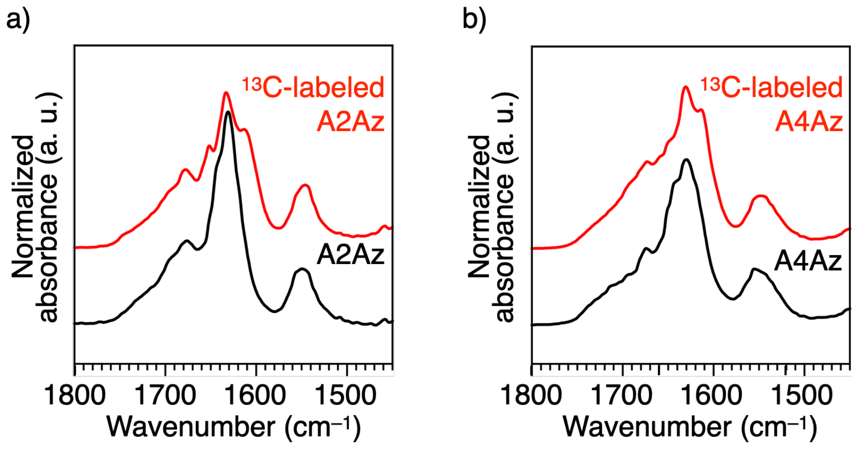

Figure S14. IR spectra of a) A2Az (1.0 wt%) and b) A4Az fibril (1.0 wt%) without (black line) and with (red line) ^{13}C label at 6th alanine residues (Ac-RAzDARADA-NH_2 and Ac-RADAzRADA-NH_2). New ^{13}C=O bands at 1613 cm^{-1} without deforming the spectral profiles of the ^{12}C=O vibration at 1626 cm^{-1} in A2Az fiber and 1622 cm^{-1} in A4Az fibril, indicate that both of them are composed of parallel β-sheet structures.[37]

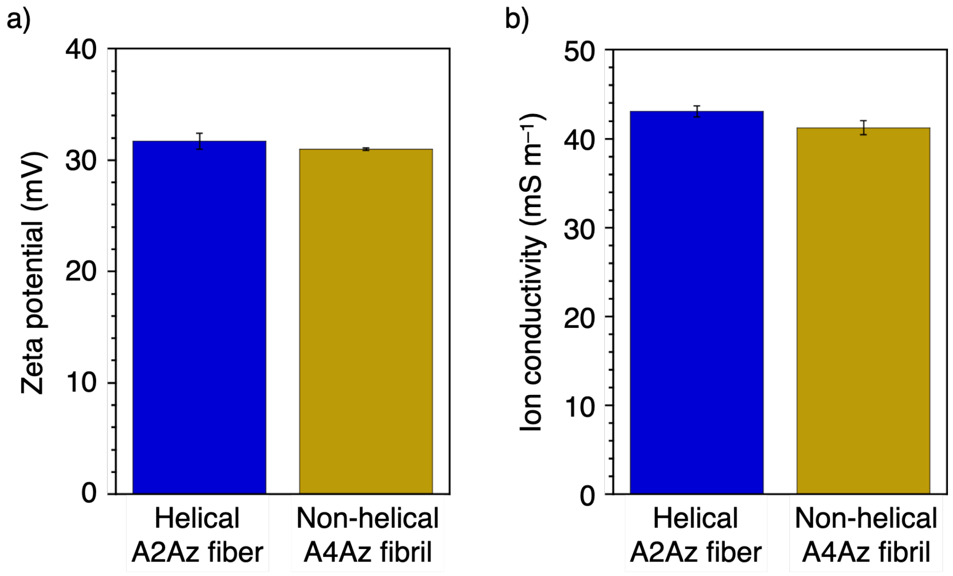

Figure S15. a) Zeta potential and b) ion conductivity of A2Az (1.0 wt%) (blue) and A4Az fibril (1.0 wt%, brown) in water containing TFA (2.2 v/v%).

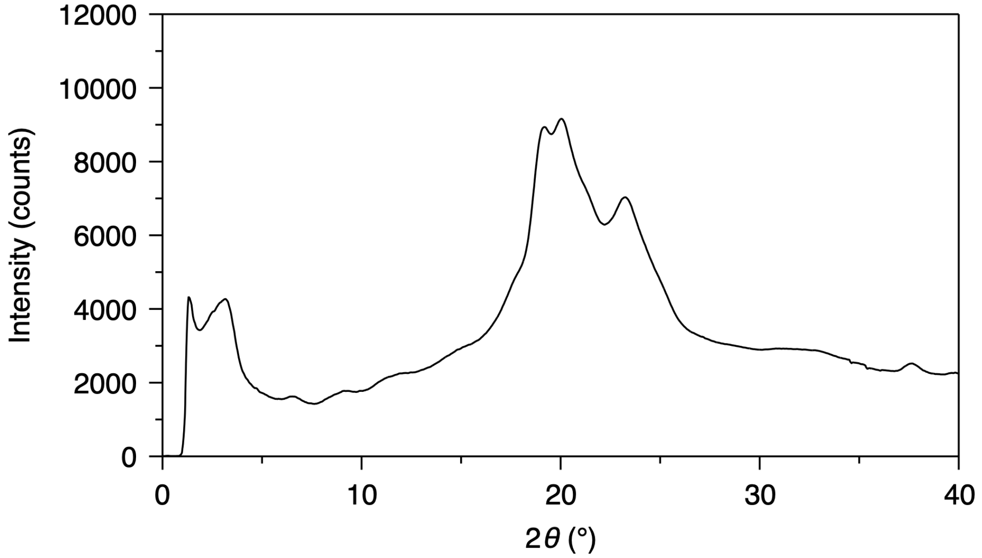

Figure S16. PXRD pattern of A2Az. Summary of PXRD data is shown in Table S1.

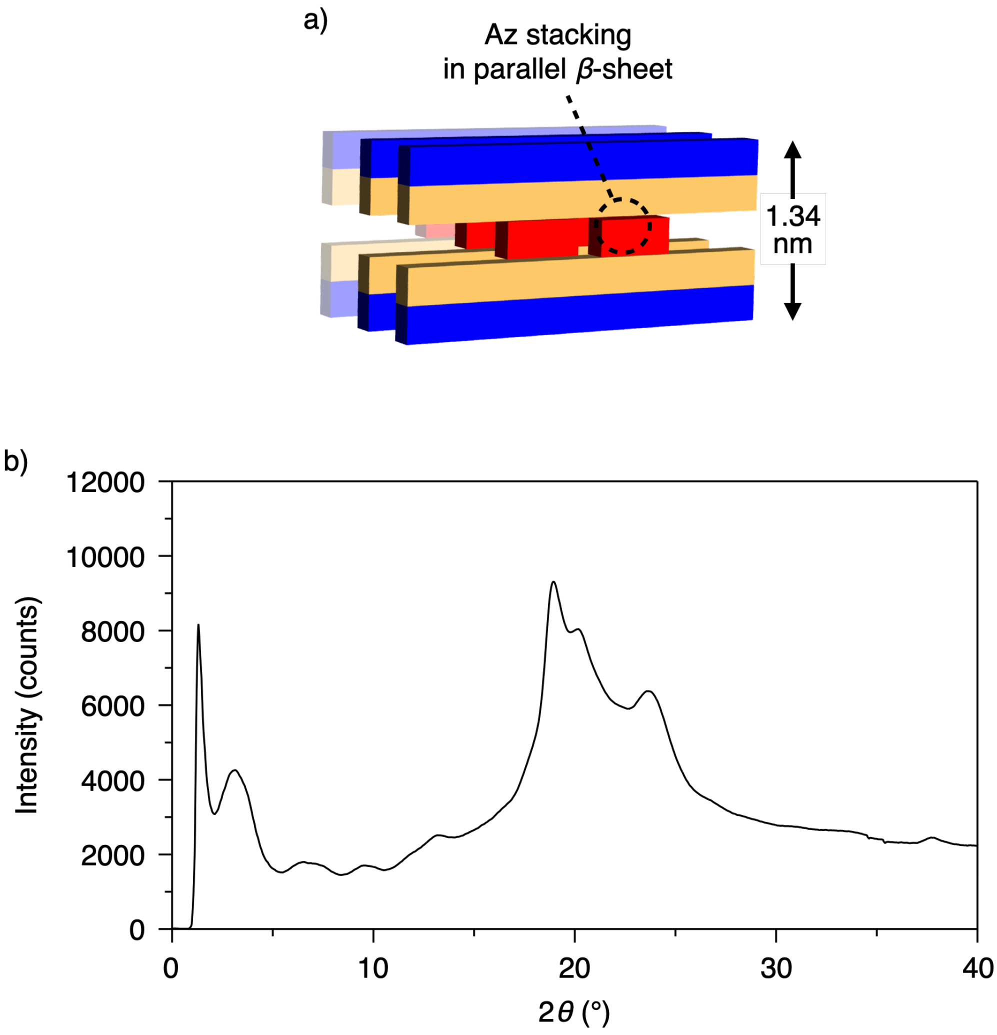

Figure S17. a) Plausible packing structure and b) PXRD pattern of A4Az. Summary of PXRD data is shown in Table S1.

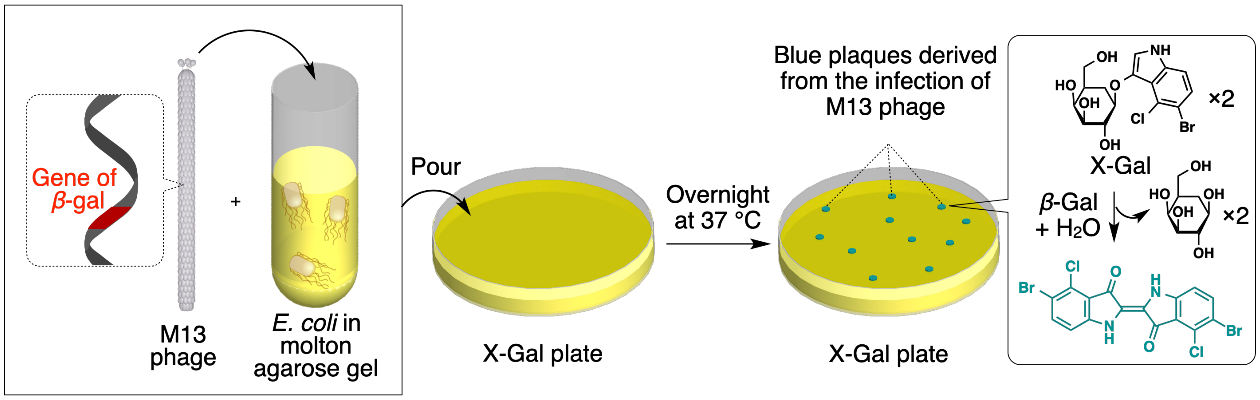

Figure S18. Schematic illustration of the plaque assay in which the amount of M13 phage is evaluated by the number of blue plaques on X-gal plate.

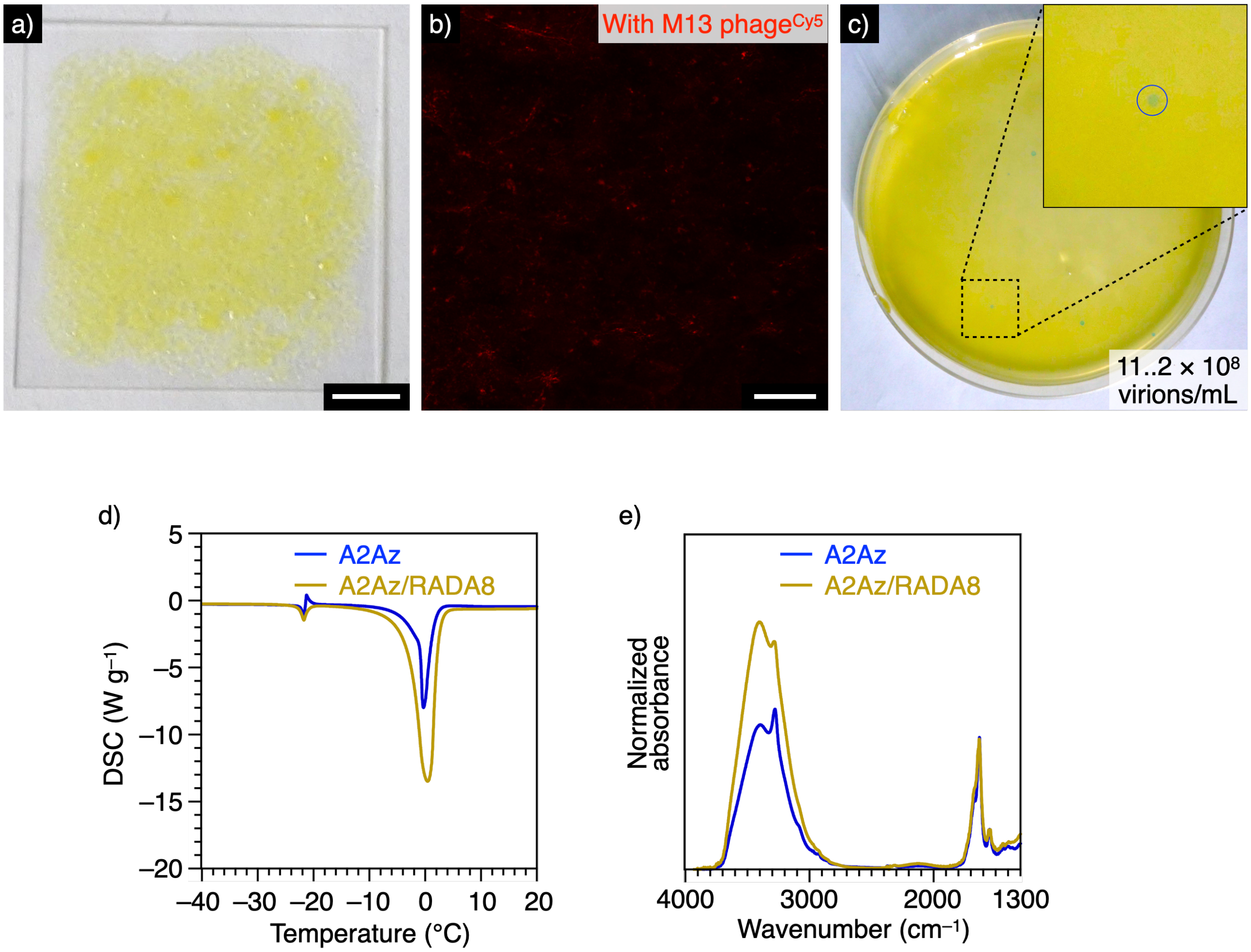

Figure S19. a) Photograph of A2Az/RADA8 -coated film (A2Az/RADA8, weight ratio 80:20). Scale bar: 3 mm. b) CLSM (λ_{ex} = 647 nm, λ_{obs} = 650–750 nm) of the glass substrates coated with A2Az/RADA8 fibril after the incubation with M13 phage^{Cy5} (17.9 × 10^{11} virions/mL). Scale bar: 100 μm. c) Photograph of X-gal plate after plaque assay for M13 phage adsorbed onto A2Az/RADA8-coated film. Inset: zoom image of the X-gal plate in which M13 phage-infected *E. coli* are indicated by a blue circle. d) DSC trace of 1.0 wt% hydrogel sample of A2Az (blue) and A2Az/RADA8 (brown). e) IR spectra of A2Az (1.0 wt%, blue) and A2Az/RADA8 (1.0 wt%, brown).

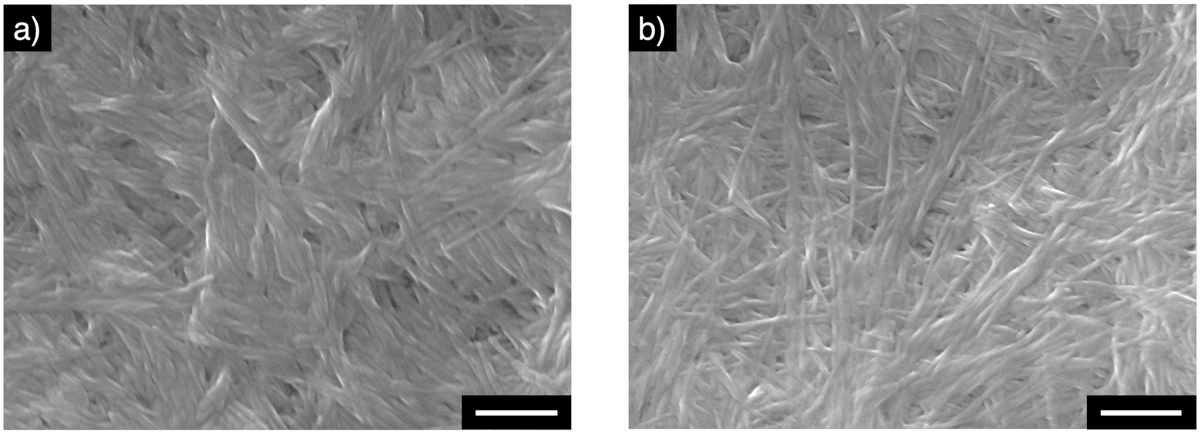

Figure S20. a, b) SEM image of A2Az fiber-coated substrate before (a) and after (b) washing with Tris-HCl buffer (1 M Tris-HCl, pH 7.5). Scale bars: 500 nm.

The SEM observations visualized the high homogeneity of the peptide assemblies before the washing process, and the distribution and morphology of the peptide assemblies were retained after washing, suggesting high stability.

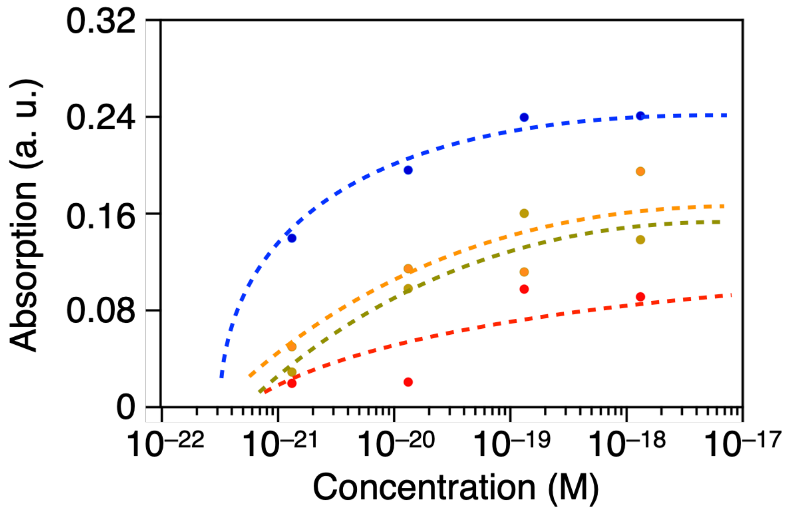

Figure S21. ELISA curves of the binding of M13 phage at different concentrations to A2Az helical (blue), A4Az non-helical (brown), RADA16 non-helical (red), and A2Az/RADA8 non-helical fibers (orange).

K_d for A2Az helical fibers was estimated to be <5×10^{-22} virions/mL, lower than those for other peptide fibers.

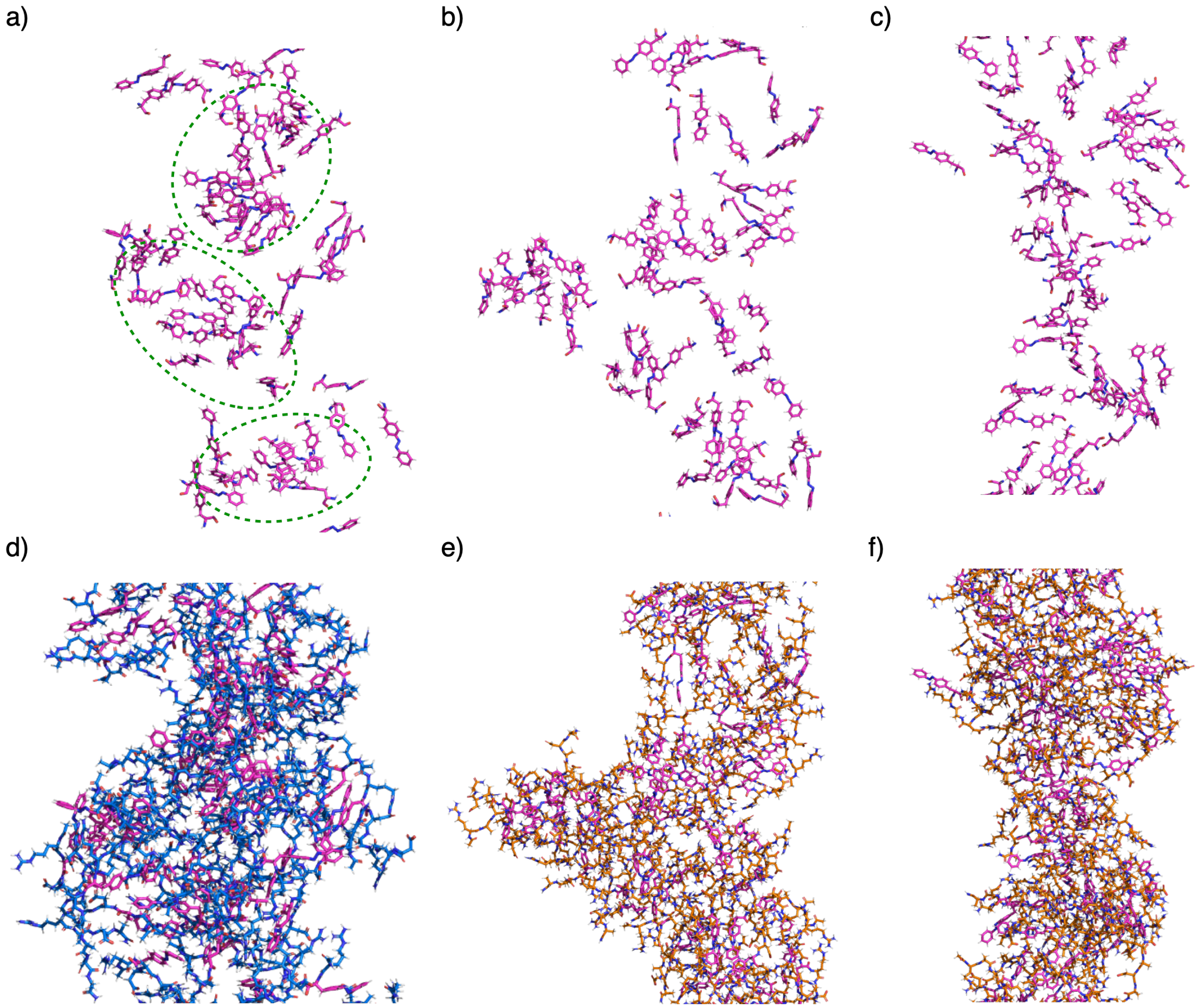

Figure S22. a–f) Snapshot of distributions of Az moieties in A2Az (a), A4Az (b), and A6Az (c) assemblies, and the original snapshot of A2Az (d), A4Az (e), and A6Az (f) assemblies in water at 300 K calculated by all-atom MD simulations. Green dashed circles in (a) indicate clustering of the Az moieties.

In the assemblies of A2Az, most of the Az moieties form stacks and aggregates. In contrast, the Az moieties in A4Az and A6Az are rather dispersed throughout the fiber. Furthermore, the orientation of the Az moieties in A2Az is mostly vertical to the direction of fiber extension, while they are more parallel to that direction in A4Az and A6Az. Based on these analyses, it is likely that

differences in the stacking formation, orientation, and aggregation of the Az moieties would result in assemblies with different morphologies.

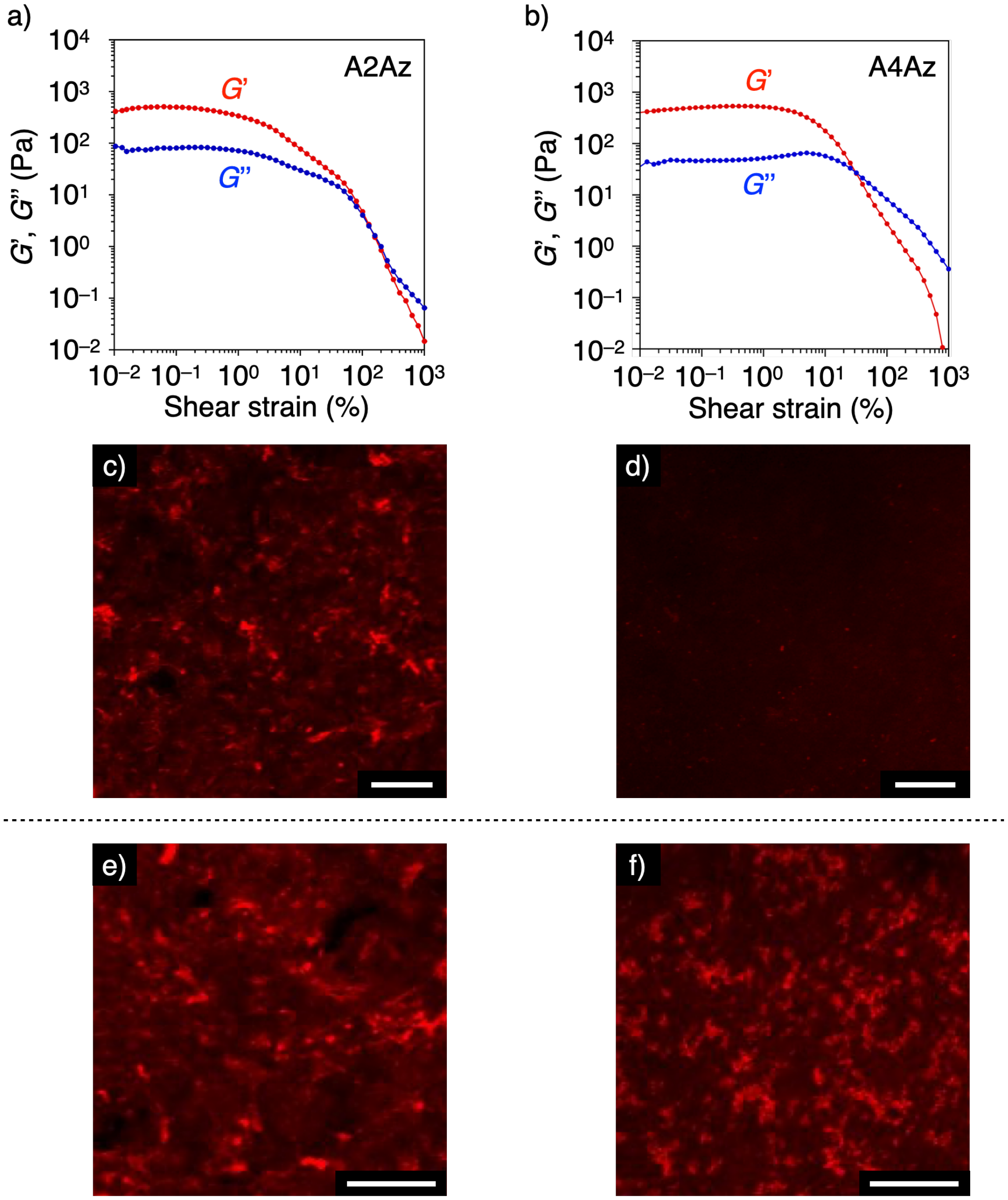

Figure S23. a, b) Strain-dependent storage moduli *G*' (red) and loss moduli *G*'' (blue) of hydrogels composed of A2Az (0.7 wt%) (a) and A4Az (0.35 wt%) (b) in water containing TFA (2.2 v/v%) at 20 °C. c, d) CLSM (λ_{ex} = 647 nm, λ_{obs} = 650–750 nm) images of M13 phage^{Cy5}-adhered glass substrates coated with A2Az (0.7 wt%) and A4Az (0.35 wt%). CLSM (λ_{ex} = 647 nm, λ_{obs} = 650–750 nm) images of M13 phage^{Cy5}-adhered glass substrates coated with A2Az (1.0 wt%) at 25 °C (e) or 37 °C (f). Scale bars: 100 µm.

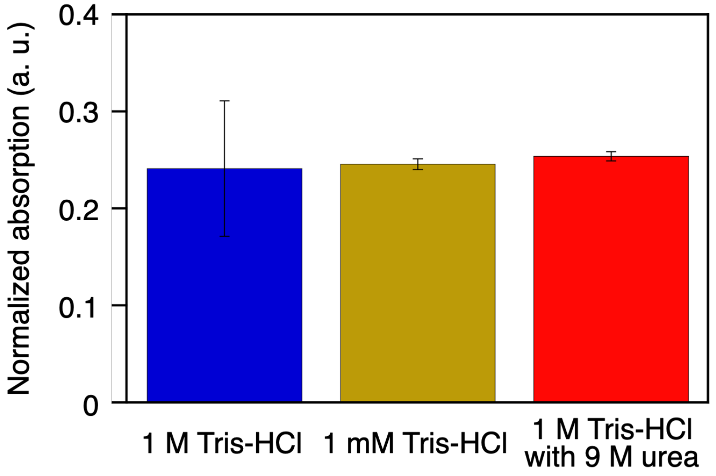

Figure S24. The amount of M13 phage adsorbed onto A2Az fiber-coated substrate under the conditions of 1 M Tris-HCl (blue), 1 mM Tris-HCl (brown), and with 9 M urea (red) as evaluated by ELISA assay.

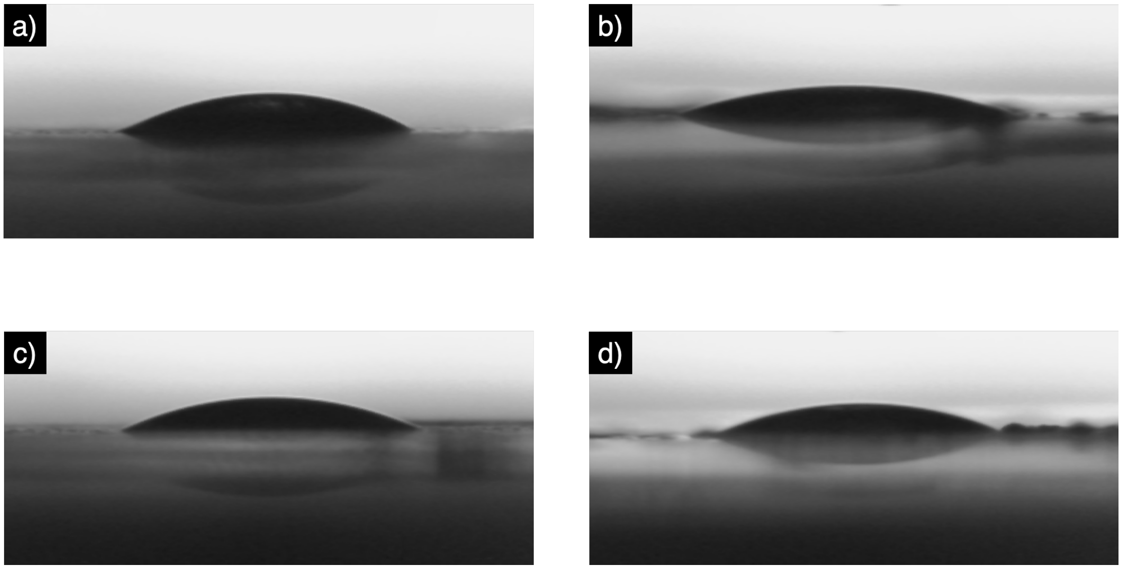

Figure S25. a–d) Photograph of water droplet on A2Az- (a), A4Az- (b), RADA16- (c), and A2Az/RADA8- (weight ratio of A2Az:RADA8 = 80:20, d) coated substrates, and their contact angles are, 30.8 ± 2.1, 20.1 ± 1.2, 23.1 ± 2.5, and 19.6 ± 3.3°, respectively.

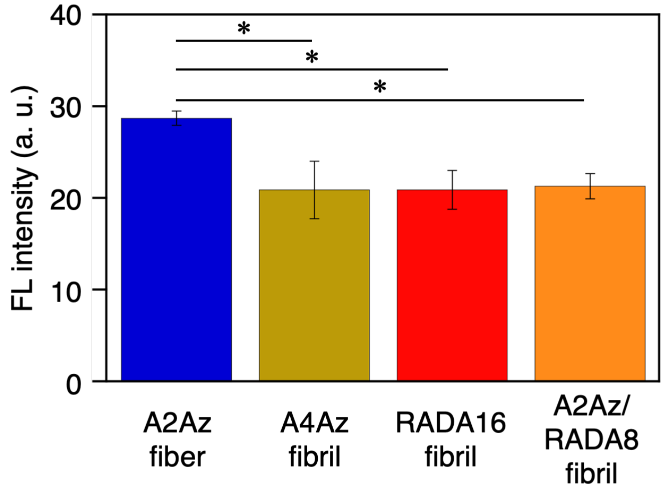

Figure S26. Fluorescence intensity of lysozyme^{Rho} (λ_{ex} = 550 nm, λ_{obs} = 580 nm) adsorbed onto glass substrates coated with A2Az (blue), A4Az (brown), RADA16 (red), and A2Az/RADA8 (weight ratio of A2Az:RADA8 = 80:20, orange) after incubation with an aqueous solution of lysozyme^{Rho} (1.6 mg/mL) for 1 min followed by rinsing. Statistical significance was examined by one-sided Student's *t* test (*p < 0.03). Bars represent mean values ± SD from three different samples.

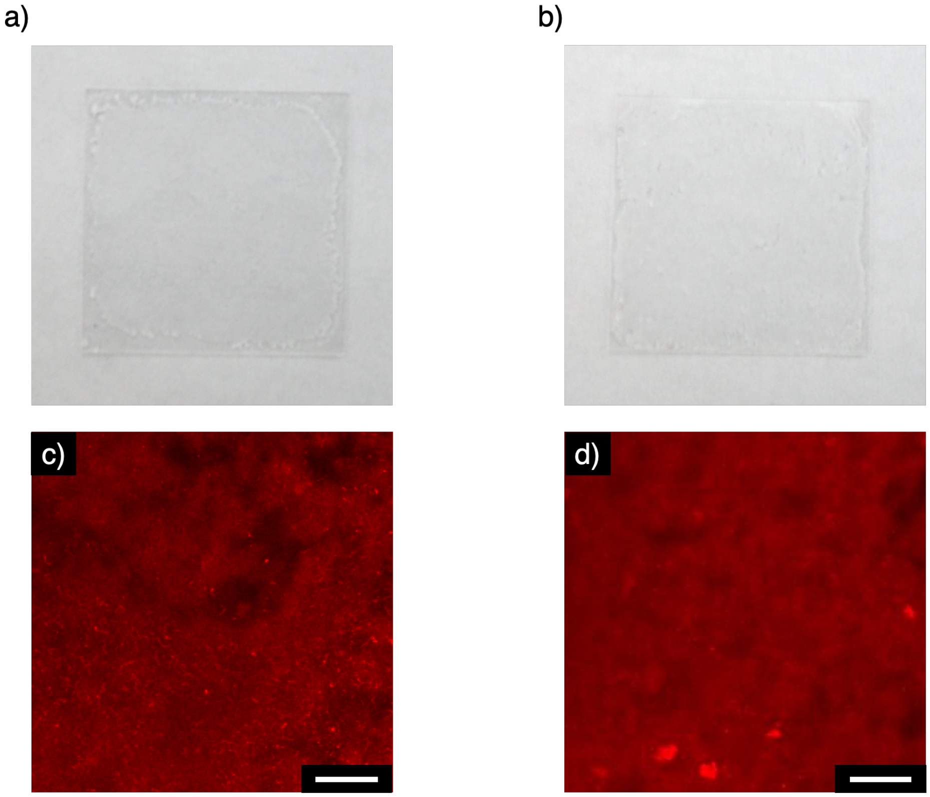

Figure S27. a, b) Photographs of glass substrates coated with naphthyl- (a) or biphenyl-introduced (b) amphiphilic peptides instead of Az group of A2Az. c, d) CLSM (λ_{ex} = 647 nm, λ_{obs} = 650–750 nm) images of M13 phage^{Cy5}-adhered glass substrates coated with naphthyl (1.0 wt%) (c) or biphenyl (1.0 wt%) (d)-introduced amphiphilic peptides instead of Az group of A2Az.

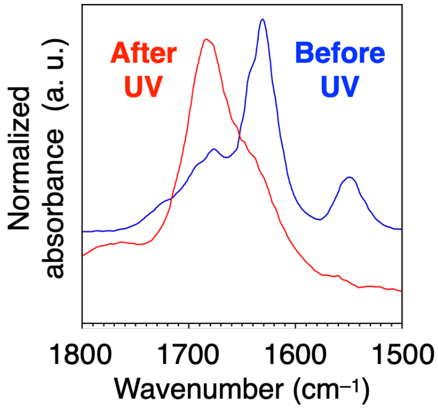

Figure S28. IR spectra of A2Az fiber (0.7 wt%) in water containing TFA (2.2 v/v%) before (blue) and after (red) 350-nm light irradiation for 40 min.

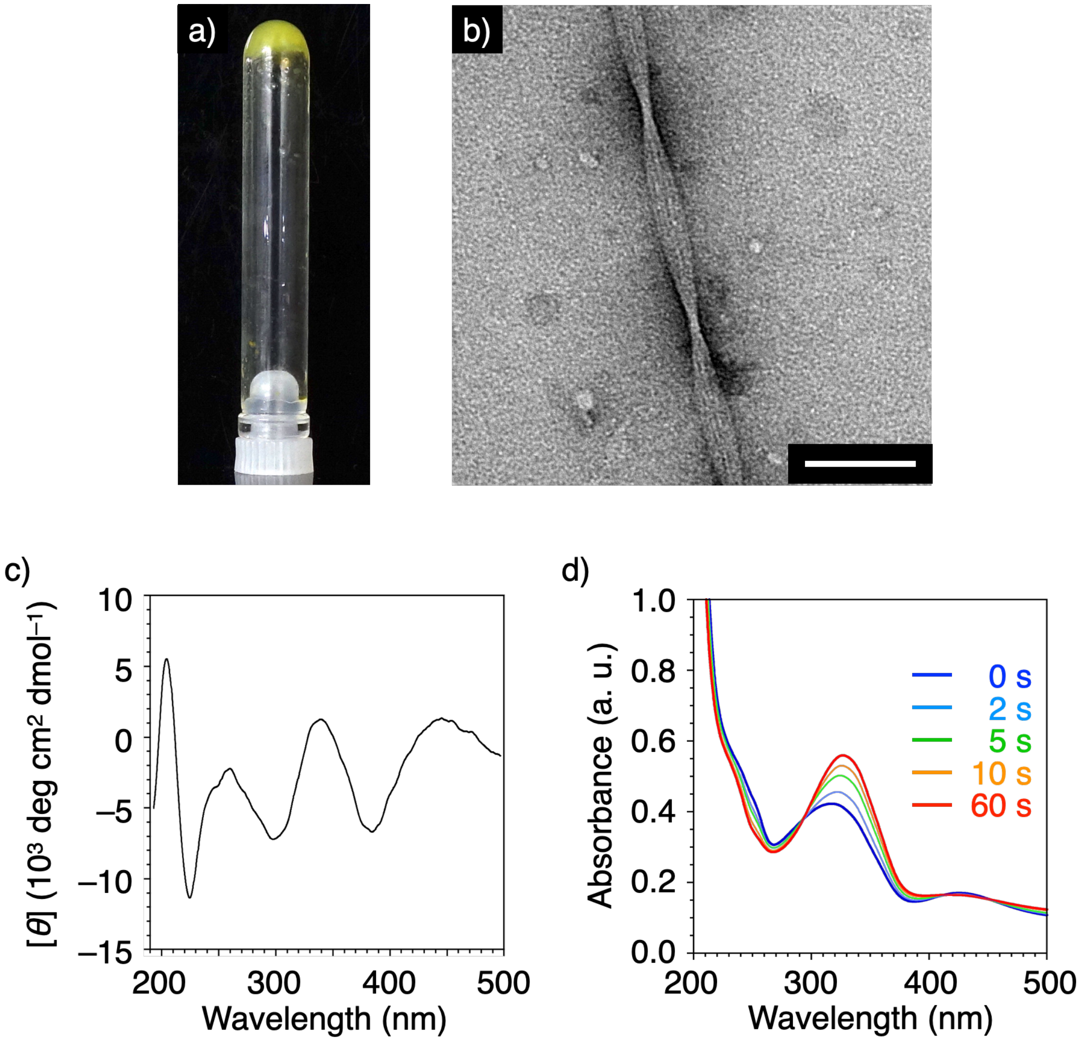

Figure S29. a) Photograph and b) TEM image of A2Az (0.7 wt%) in water containing TFA (2.2 v/v%) after 450-nm light irradiation for 60 min following 350-nm light irradiation for 60 min. Scale bar: 100 nm. The sample was stained with uranyl acetate. c) CD spectrum of A2Az (0.7 wt%) in water containing TFA (2.2 v/v%) after 450-nm light irradiation for 60 min following 350-nm light irradiation for 60 min. d) UV-Vis absorption spectral change of A2Az (0.7 wt%) after 350-nm light irradiation for 5 min followed by 450-nm light irradiation for 0 s (blue), 2 s (light blue), 5 s (green), 10 s (orange), and 60 s (red) in water containing TFA (2.2 v/v%).

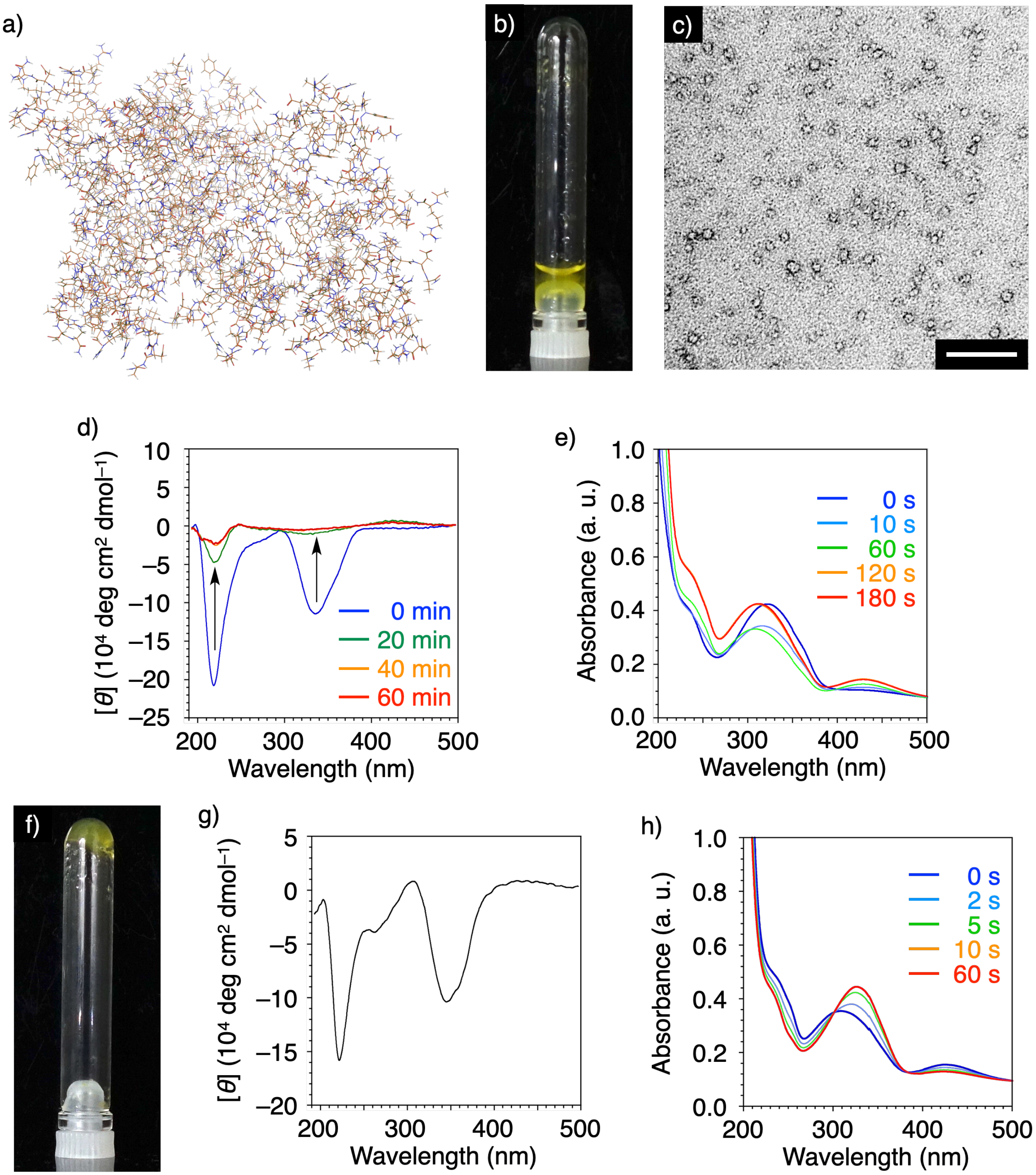
a)
b)
c)
d)
10
5
0
−5
−10
−15
−20
−25
[θ] (10⁴ deg cm² dmol⁻¹)
200
300
400
500
Wavelength (nm)
0 min
20 min
40 min
60 min
e)
1.0
0.8
0.6
0.4
0.2
0.0
Absorbance (a. u.)
200
300
400
500
Wavelength (nm)
0 s
10 s
60 s
120 s
180 s
f)
g)
5
0
−5
−10
−15
−20
[θ] (10⁴ deg cm² dmol⁻¹)
200
300
400
500
Wavelength (nm)
h)
1.0
0.8
0.6
0.4
0.2
0.0
Absorbance (a. u.)
200
300
400
500
Wavelength (nm)
0 s
2 s
5 s
10 s
60 s

Figure S30. a) Snapshot of supramolecular structure of A4Az containing *cis*-form of Az group in water at 300 K calculated by all-atom MD simulation. b) Photograph and c) TEM image of A4Az (0.5 wt%) in water containing TFA (2.2 v/v%) after 350-nm light irradiation for 20 min. Scale bar: 100 nm. The TEM sample was stained with uranyl acetate. d) CD spectral change of A4Az (0.5 wt%) in water containing TFA (2.2 v/v%) upon 350-nm light irradiation for 0 (blue), 20 (green), 40 (orange), and 60 (red) min. e) UV-Vis absorption spectral change of A4Az (0.5 wt%) upon 350-nm light irradiation for 0 s (blue), 10 s (light blue), 60 s (green), 120 s (orange), and 180 s (red) in water containing TFA (2.2 v/v%). f) Photograph of A4Az (0.5 wt%) in water containing TFA (2.2 v/v%) after 450-nm light irradiation for 60 min following 350-nm light irradiation for 60 min. g) CD spectrum of A4Az (0.5 wt%) in water containing TFA (2.2 v/v%) after 450-nm light irradiation for 60 min following 350-nm light irradiation for 60 min. h) UV-Vis absorption spectral change of A4Az (0.5 wt%) after 350-nm light irradiation for 5 min followed by 450-nm light irradiation for 0 s (blue), 2 s (light blue), 5 s (green), 10 s (orange), and 60 s (red) in water containing TFA (2.2 v/v%).

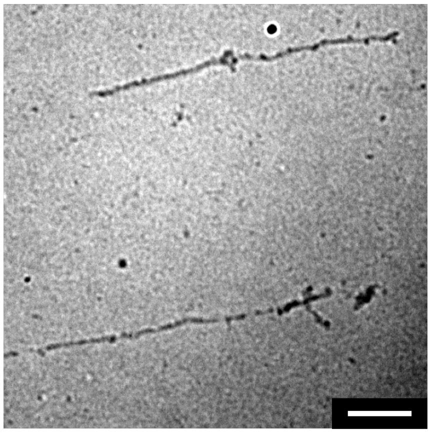

Figure S31. TEM image of the M13 phage^{Cy5}/A2Az hybrid irradiated with 350-nm light for 15 min. Scale bar: 200 nm.

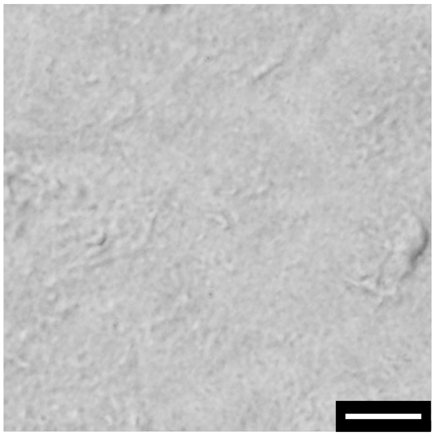

Figure S32. Phase-contrast image of A2Az-coated glass substrate containing M13 phage. The sample was prepared by mixing A2Az, M13 phage, and gold nanoparticles. After being cast on the glass substrate, the sample was rinsed. The image indicates that gold nanoparticles are hardy adsorbed. Scale bar: 20 μm.

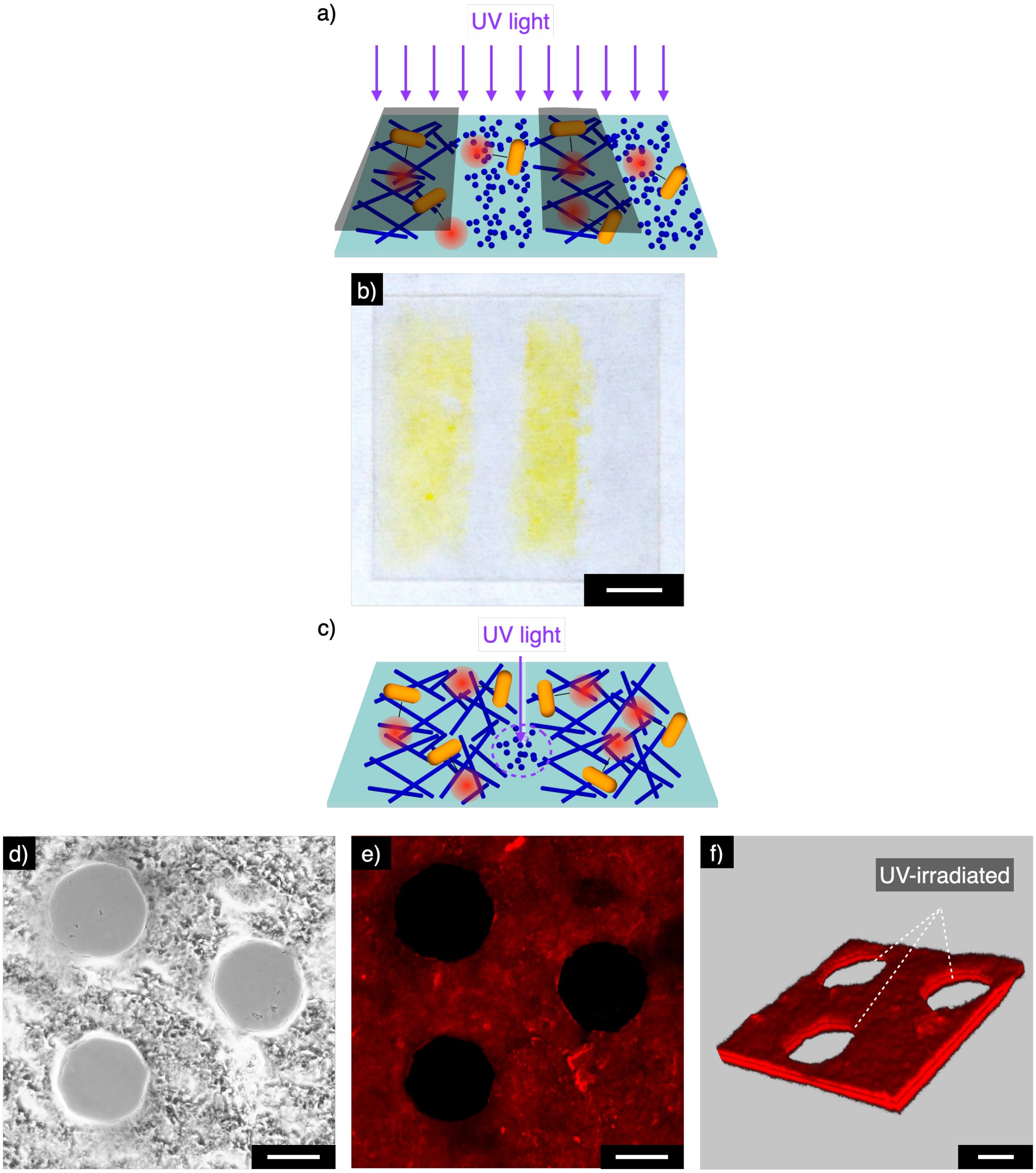

Figure S33. a,b) Schematic illustration (a) and photograph (b) of UV-irradiated M13 phage^{Cy5}-adhered glass substrate masked with stripe-shaped aluminum foil. Scale bar: 3 mm. c) Schematic illustration of photo-induce micropatterning of M13 phage^{Cy5}-adhered glass substrate. d–f) Phase-contrast (d), 2D CLSM (e), and 3D CLSM (f) images of M13 phage^{Cy5}-adhered glass substrate irradiated with UV light with a diameter of 130 μm. Scale bars: 100 μm.

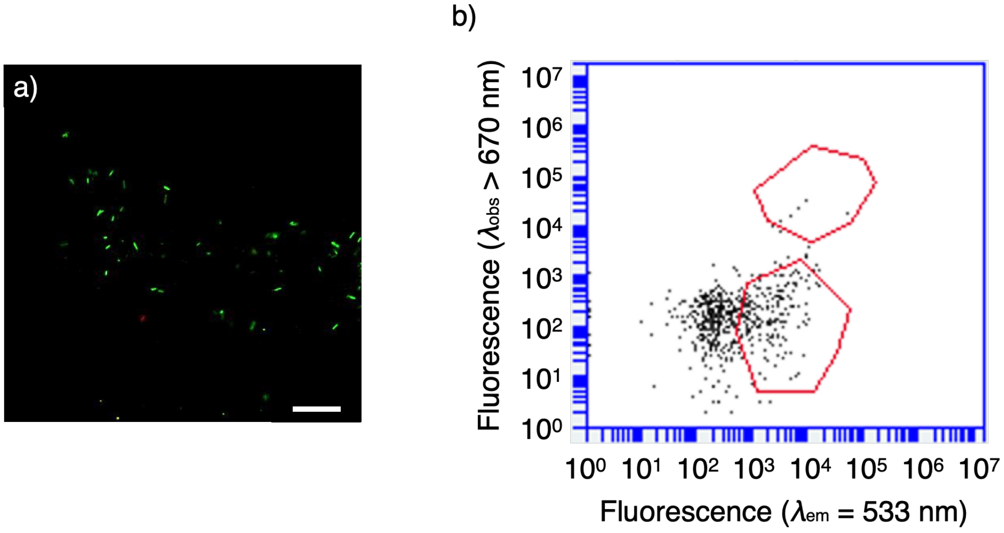

Figure S34. a) Merged CLSM images (green, λ_{ex} = 488 nm for live cells and red, λ_{ex} = 561 nm for dead cells) of *E. coli* cells in A2Az (1 wt%) hydrogel containing LB medium and LIVE/DEAD™ *Bac*Light™ Bacterial Viability and Counting Kit. Scale bar: 20 µm. b) A representative plots of green fluorescence (λ_{ex} = 488 nm, λ_{obs} = 533 nm) and red fluorescence (λ_{ex} = 488 nm, λ_{obs} > 670 nm) in flow cytometry analysis. Upper and lower surround areas correspond to the plots of dead and live *E. coli* cells, respectively. Cell viability was calculated to be 96.5 ± 0.3%.

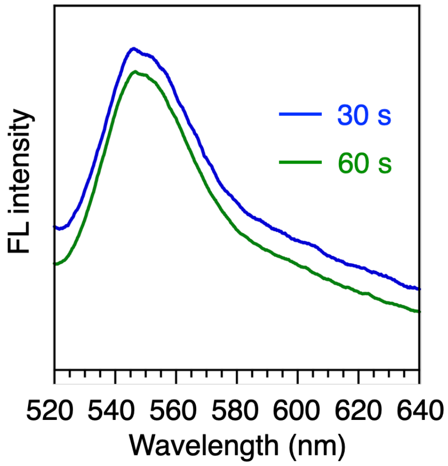

Figure S35. Fluorescent spectra (λ_{ex} = 500 nm) of A2Az hydrogel containing M13 phage (1.3 × 10^{11} virions/mL), *E. coli* cell, and SPiDER-*β*Gal (0.6 μg/mL) after UV light irradiation (λ = 350 nm) for 30 s (blue) or 60 s (green) followed by incubation for 7 days at 37 °C.

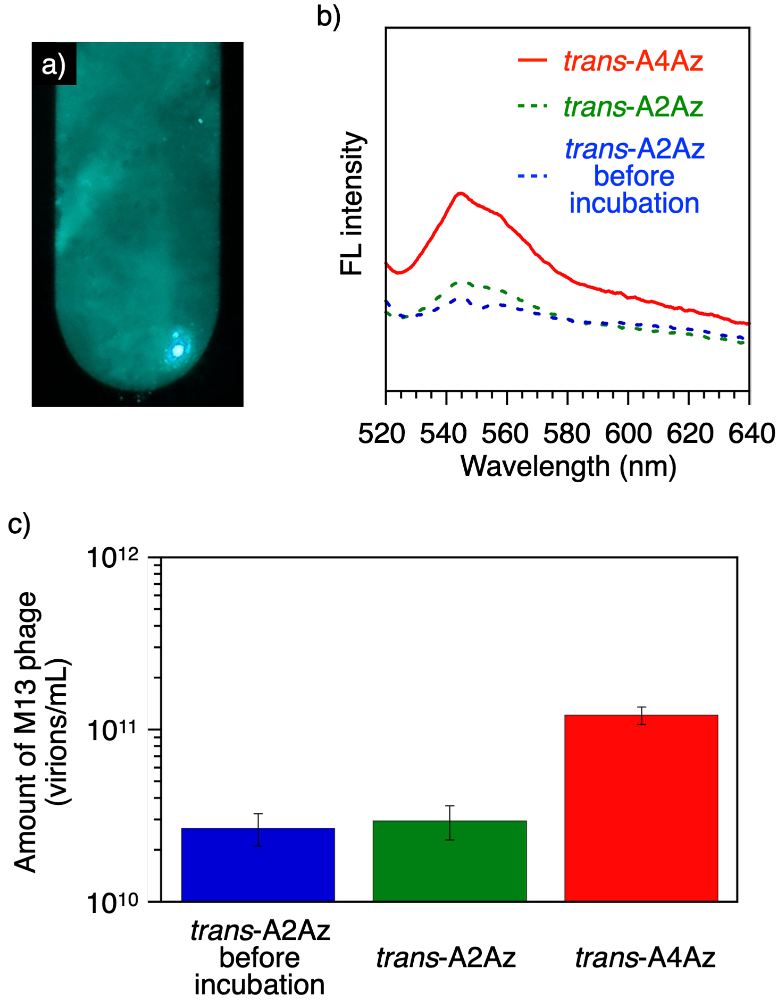

Figure S36. a) Photograph of A4Az fibril (0.5 wt%) hydrogel containing M13 phage (1.3 × 10^{11} virions/mL), *E. coli* cell, and SPiDER-*β*Gal (0.6 µg/mL) under 500-nm excitation light after incubation for 7 days at 37 °C. b) Fluorescent spectra (λ_{ex} = 500 nm) of A4Az hydrogel (red line curve), and A2Az hydrogels containing M13 phage (1.3 × 10^{11} virions/mL), *E. coli* cell, and SPiDER-*β*Gal (0.6 µg/mL) before (dashed blue line) and after (dashed green line) incubation for 7 days at 37 °C. c) The amount of M13 phage as evaluated by the plaque assay in A2Az hydrogel without (blue) and with (green) the 7-days incubation, and A4Az hydrogel with the incubation (red).

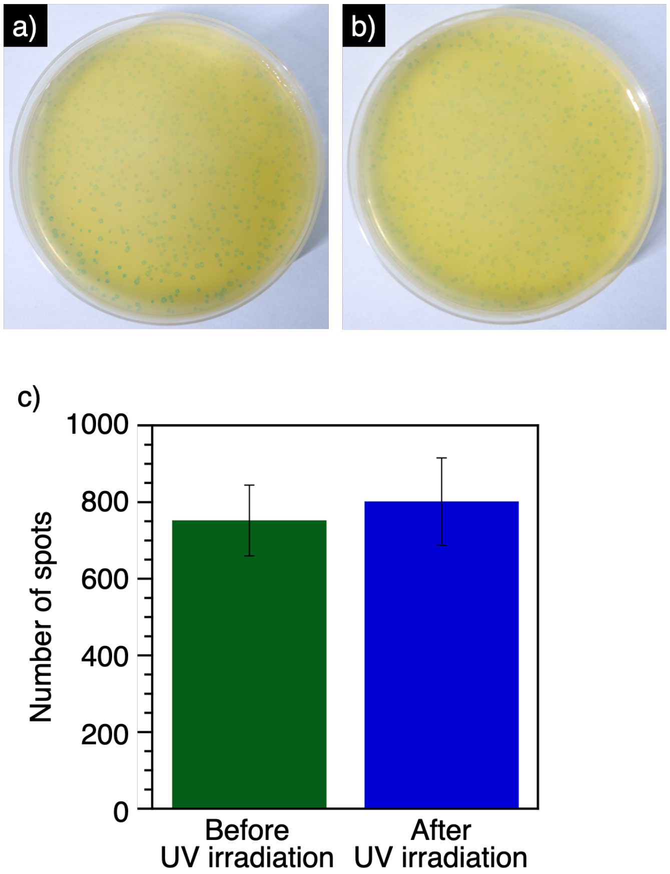

Figure S37. a–c) Photograph of X-gal plate after plaque assay for M13 phage (1.3×10^{11} virions/mL) before (a) and after (b) 350-nm irradiation for 30 s. c) The number of blue spots on X-gal plate before (green) and after (blue) the UV irradiation.

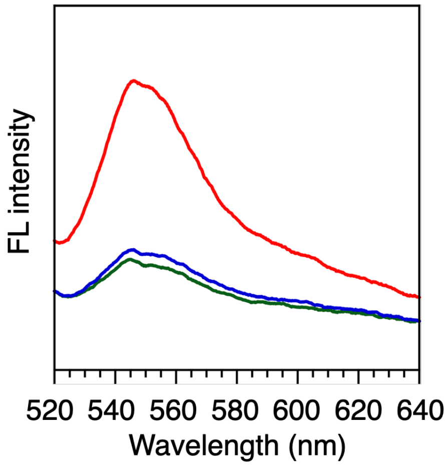

Figure S38. Fluorescent spectra (λ_{ex} = 500 nm) of A2Az hydrogels containing M13 phage (1.3 × 10^{11} virions/mL), *E. coli* cell, and SPiDER-*β*Gal (0.6 μg/mL) before (green line) and after the irradiation of 350 nm light (30 s, Xe lamp, red line), followed by the irradiation of 450 nm light (60 s, Xe lamp, blue line) to reconstruct A2Az fibers. The samples were incubated for 7 days at 37 °C.

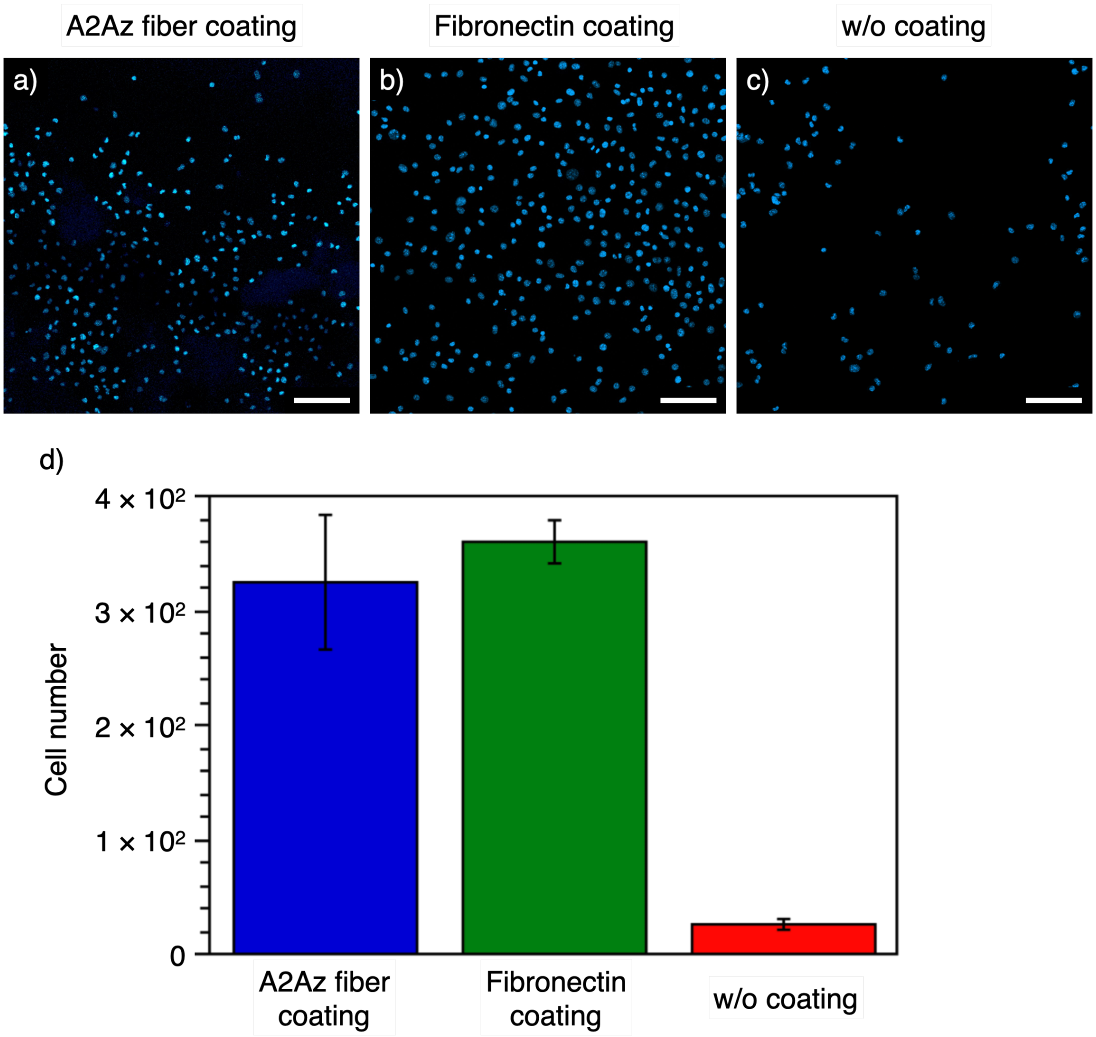

Figure S39. Fluorescence micrographs of fibroblasts attached on a glass surface coated with a) A2Az fiber, b) cell adhesive fibronectin as positive control, and c) without coating as negative control. Fibroblasts were stained with DAPI. Scale bars: 50 µm. d) The number of fibroblasts attached on glass substrate coated with A2Az fiber (blue), fibronectin (green), and without coating (red).

6. Supporting Table

	2θ (°)	$d_{obs.}$ (nm)	$d_{calc.}$ (nm)	*hkl*
A2Az	3.15<br>11.9<br>20.04<br>23.25<br>31.20	2.805<br>0.74<br>0.443<br>0.383<br>0.287	– [a]<br>0.743<br>0.444<br>0.382<br>0.287	– [a]<br>020<br>210<br>211<br>212
A4Az	3.14<br>13.18<br>20.18<br>23.65	2.814<br>0.672<br>0.440<br>0.376	– [a]<br>0.672<br>0.435<br>0.380	– [a]<br>002<br>210<br>211

Table S1. Summary of the PXRD data of A2Az, A4Az, and A6Az. $d_{obs.}$ and $d_{calc.}$ indicate distance observed from PXRD profiles and distance used for the determination of the lattice parameters, respectively. Thickness of the self-assemblies of A2Az and A4Az were 1.486 and 1.344, respectively. Width of the self-assemblies of A2Az and A4Az were 1.506 and 1.560, respectively. Distance along the fiber direction in A2Az and A4Az were 0.930 and 0.920, respectively.

[a] Layer distance (chain direction) of stacked β-sheets or superstructure composed of molecular assemblies.

7. References

[17]. A. Yaguchi, M. Oshikawa, G. Watanabe, H. Hiramatsu, N. Uchida, C. Hara, N. Kaneko, K. Sawamoto, T. Muraoka, I. Ajioka, *Nat. Commun.* **2021**, *12*, 6623.

[18]. A. Yaguchi, H. Hiramatsu, A. Ishida, M. Oshikawa, I. Ajioka, T. Muraoka, *Chem. Eur. J.* **2021**, *27*, 9295–9301.

[19]. B. Hess, C. Kutzner, D. van der Spoel, E. Lindahl, *J. Chem. Theory Comput.* **2008**, *4*, 435-447.

[20]. K. Lindorff-Larsen, S. Piana, K. Palmo, P. Maragakis, J. L. Klepeis, R. O. Dror, D. E. Shaw, *Proteins* **2010**, *78*, 1950–1958.

[21]. G. Bussi, D. Donadio, M. Parrinello, *J. Chem. Phys.* **2007**, *126*, 14101.

[22]. H. J. C. Berendsen, J. P. M. Postma, W. F. van Gunsteren, A. DiNola, J. R. Haak, *J. Chem. Phys.* **1984**, *81*, 3684–3690.

[23]. S. Nosé, *Mol. Phys.* **1984**, *52*, 255–268.

[24]. S. Nosé, S. *J. Chem. Phys.* **1984**, *81*, 511–519.

[25]. W. G. Hoover, *Phys. Rev. A* **1985**, *31*, 1695–1697.

[26]. M. Parrinello, A. Rahman, *J. Appl. Phys.* **1981**, *52*, 7182–7190.

[27]. B. Hess, H. Bekker, H. J. C. Berendsen, J. G. E. M. Fraaije, *J. Comput. Chem.* **1997**, *18*, 1463–1472.

[28]. U. Essmann, L. Perera, M. L. Berkowitz, T. Darden, H. Lee, L. G. Pedersen, *J. Chem. Phys.* **1995**, *103*, 8577–8593.

[29]. C. C. Liu, A. V. Mack, M.-L. Tsao, J. H. Mills, H. S. Lee, H. Choe, M. Farzan, P. G. Schultz, V. V. Smider, *Proc. Natl. Acad. Sci. U. S. A.* **2008**, *105*, 17688–17693.

[30]. T. Maniatis, E. F. Fritsch, J. Sambrook, *Molecular cloning: a laboratory manual*, Cold Spring Harbor Laboratory, New York **1982**.

[31]. W.-J. Yanga, D. Shiuanb, *J. Immunol. Methods* **2003**, *276*, 175–183.

[32]. N. Uchida, Y. Ryu, Y. Takagi, K. Yoshizawa, K. Suzuki, Y. Anraku, I. Ajioka, N. Shimokawa, M. Takagi, N. Hoshino, T. Akutagawa, T. Matsubara, T. Sato, Y. Higuchi, H. Ito, M. Morita, T. Muraoka, *J. Am. Chem. Soc.* **2023**, *145*, 6210–6220.

[33]. D. I. Staquicini, F. H. F. Tang, C. Markosian, V. J. Yao, F. I. Staquicini, E. Dodero-Rojasc, V. G. Contessoto, D. Davis, P. O'Brien, N. Habib, T. L. Smith, N. Bruiners, R. L. Sidman, M. L. Gennaro, E. C. Lattime, S. K. Libutti, P. C. Whitfordi, S. K. Burley, J. N. Onuchic, W. Arap, R. Pasqualini, *Proc. Natl. Acad. Sci. USA* **2021**, *118*, e2105739118.

[34]. Y. Huang, C.-Y. Chiang, S. K. Lee, Y. Gao, E. L. Hu, J. D. Yoreo, A. M. Belcher, *Nano Lett.* **2005**, *5*, 1429‑1434.

[35]. K. A. Noren, C. J. Noren, *Methods* **2001**, *23*, 169‑178.

[36]. A. Yaguchi, H. Hiramatsu, A. Ishida, M. Oshikawa, I. Ajioka, T. Muraoka, *Chem. Eur. J.* **2021**, *27*, 9295–9301.

[37]. H. Hiramatsu, M. Lu, Y. Goto, T. Kitagawa, *Bull. Chem. Soc. Jpn.* **2010**, *83*, 495–504.
